# Subacromial decompression versus diagnostic arthroscopy for shoulder impingement: randomised, placebo surgery controlled clinical trial

**DOI:** 10.1136/bmj.k2860

**Published:** 2018-07-19

**Authors:** Mika Paavola, Antti Malmivaara, Simo Taimela, Kari Kanto, Jari Inkinen, Juha Kalske, Ilkka Sinisaari, Vesa Savolainen, Jonas Ranstam, Teppo L N Järvinen

**Affiliations:** 1 Department of Orthopedics and Traumatology, Helsinki University Hospital, Töölö hospital, Helsinki, Finland; 2 National Institute for Health and Welfare, Centre for Health and Social Economics, Helsinki, Finland; 3 Finnish Centre for Evidence-Based Orthopedics (FICEBO), Department of Orthopedics and Traumatology, University of Helsinki, Helsinki, Finland; 4 Department of Orthopedics and Traumatology, Tampere University Hospital, TAYS Hatanpää, Tampere, Finland; 5 Fysios Finlayson, Physiotherapy Centre Kunnon Klinikka Oy, Tampere, Finland; 6 Department of Orthopedics and Traumatology, Helsinki University Hospital, Jorvi Hospital, Espoo, Finland; 7 Terveystalo, Helsinki, Finland; 8 Pohjola Hospital, Helsinki, Finland; 9 Department of Clinical Sciences Lund, Orthopedics, Lund University, Lund, Sweden

## Abstract

**Objective:**

To assess the efficacy of arthroscopic subacromial decompression (ASD) by comparing it with diagnostic arthroscopy, a placebo surgical intervention, and with a non-operative alternative, exercise therapy, in a more pragmatic setting.

**Design:**

Multicentre, three group, randomised, double blind, sham controlled trial.

**Setting:**

Orthopaedic departments at three public hospitals in Finland.

**Participants:**

210 patients with symptoms consistent with shoulder impingement syndrome, enrolled from 1 February 2005 with two year follow-up completed by 25 June 2015.

**Interventions:**

ASD, diagnostic arthroscopy (placebo control), and exercise therapy.

**Main outcome measures:**

Shoulder pain at rest and on arm activity (visual analogue scale (VAS) from 0 to 100, with 0 denoting no pain), at 24 months. The threshold for minimal clinically important difference was set at 15.

**Results:**

In the primary intention to treat analysis (ASD versus diagnostic arthroscopy), no clinically relevant between group differences were seen in the two primary outcomes at 24 months (mean change for ASD 36.0 at rest and 55.4 on activity; for diagnostic arthroscopy 31.4 at rest and 47.5 on activity). The observed mean difference between groups (ASD minus diagnostic arthroscopy) in pain VAS were −4.6 (95% confidence interval −11.3 to 2.1) points (P=0.18) at rest and −9.0 (−18.1 to 0.2) points (P=0.054) on arm activity. No between group differences were seen between the ASD and diagnostic arthroscopy groups in the secondary outcomes or adverse events. In the secondary comparison (ASD versus exercise therapy), statistically significant differences were found in favour of ASD in the two primary outcomes at 24 months in both VAS at rest (−7.5, −14.0 to −1.0, points; P=0.023) and VAS on arm activity (−12.0, −20.9 to −3.2, points; P=0.008), but the mean differences between groups did not exceed the pre-specified minimal clinically important difference. Of note, this ASD versus exercise therapy comparison is not only confounded by lack of blinding but also likely to be biased in favour of ASD owing to the selective removal of patients with likely poor outcome from the ASD group, without comparable exclusions from the exercise therapy group.

**Conclusions:**

In this controlled trial involving patients with a shoulder impingement syndrome, arthroscopic subacromial decompression provided no benefit over diagnostic arthroscopy at 24 months.

**Trial registration:**

Clinicaltrials.gov NCT00428870.

**Figure:**
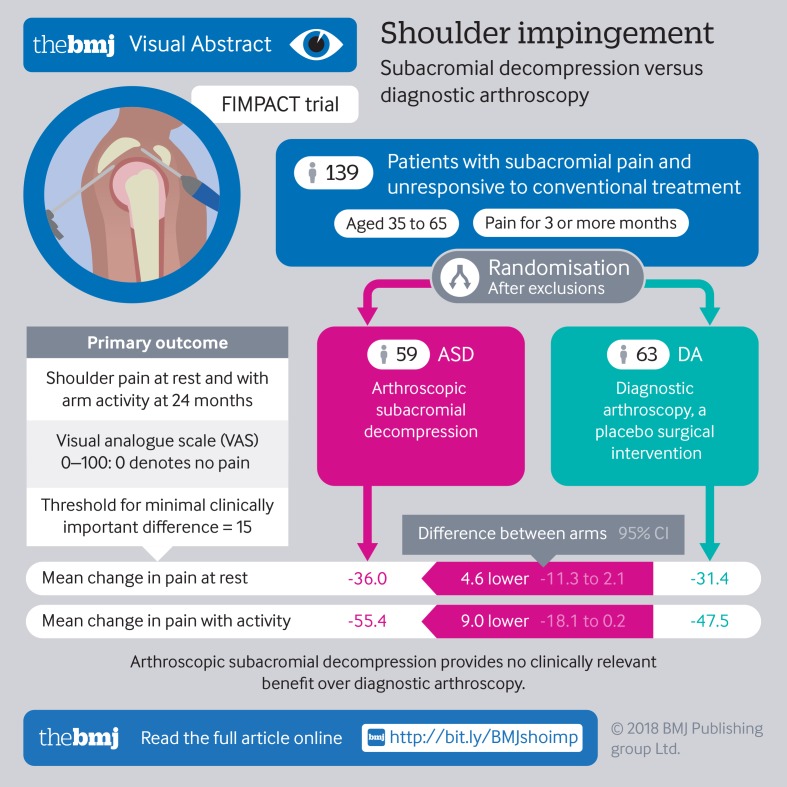


## Introduction

Management of shoulder pain has been estimated to account for 4.5 million visits to physicians and $3bn (£2.3bn; €2.6bn) financial burden each year in the US alone.^1^ ^2^ As 44-70% of patients with shoulder pain are diagnosed as having a shoulder impingement syndrome, annual direct medical costs of this complaint are estimated at more than $1bn in the US.^3^ ^4^ ^5^ The pathognomonic clinical sign of shoulder impingement syndrome, subacromial shoulder pain while lifting the arm, is commonly attributed to “impingement” of the rotator cuff tendons between the humeral head and the overlying acromion. Premised on this rationale, the undersurface of the acromion is smoothened to decompress the passage of the rotator cuff tendon through the subacromial space in a surgical procedure called subacromial decompression. Although various non-operative treatment modalities are recommended as initial treatment for patients with shoulder impingement symptoms,^6^ ^7^ subacromial decompression has become one of the most frequently performed orthopaedic procedures in the world.^8^ With the advent of arthroscopy, the number of subacromial decompression procedures has increased many times between the 1980s and the 2010s.^9^ ^10^ We conducted a multicentre, randomised, double blind, placebo surgery controlled trial to assess the efficacy of arthroscopic subacromial decompression (ASD) in patients with shoulder symptoms consistent with shoulder impingement syndrome.

## Methods

### Trial design

We conducted this superiority trial at three orthopaedic clinics in Finland from 1 February 2005 to 25 June 2015. Details of the trial design and conduct have been published elsewhere.^11^ The patients, the people who collected and analysed the data, and those who interpreted the principal findings for the ASD versus diagnostic arthroscopy comparison (see below, “Blinded data interpretation”) were unaware of the study group assignments. On entering the study, patients were unequivocally informed that they might undergo diagnostic arthroscopy and that they would be allowed to consider crossing over to ASD if they did not have adequate relief of symptoms, preferably no sooner than six months after randomisation.

### Participants

We enrolled patients aged 35-65 years who had subacromial pain (for more than three months) that was unresponsive to conventional conservative treatment and had clinical findings consistent with shoulder impingement syndrome. All patients had magnetic resonance imaging with intra-articular contrast to exclude a rotator cuff tear. Detailed inclusion and exclusion criteria are provided in table S1 in appendix 2.

### Randomisation and blinding

In an attempt to obtain three balanced study groups of similar size, we planned a twofold, sequential randomisation as follows. Firstly, during the baseline appointment, patients were randomised to surgical or conservative treatment (exercise therapy) in a 2:1 ratio. Patients randomised to exercise therapy started standardised physiotherapy within two weeks of the baseline appointment, whereas those allocated to surgery were scheduled for surgery with the aim of carrying out the procedure within 12 weeks of this first randomisation. In patients allocated to surgery, we did a diagnostic arthroscopy to rule out a rotator cuff tendon tear and other obvious intra-articular pathology needing surgical treatment. If we found a full or a partial thickness rotator cuff tear large enough to need repair (grade III) according to clinical practice guidelines,^12^ we excluded the patient and repaired the tear. Patients with a partial tear that did not need repair (grade I and II) were included in the study. If the eligibility of the patient was confirmed in diagnostic arthroscopy, the surgeon asked a research nurse to carry out the second randomisation by opening an envelope containing the study group assignment (ASD or diagnostic arthroscopy; ratio 1:1). Only the orthopaedic surgeon and other staff in the operating room were made aware of the surgical group assignment, and they did not participate in further treatment or follow-up of the patient.

Randomisation was carried out using sequentially numbered sealed opaque envelopes. Separate randomisation lists for each of the three centres, with blocks varying randomly in size, were prepared by a statistician with no involvement in the clinical care of participants in the trial.

### Study interventions

#### Exercise therapy

Supervised, progressive, individually designed physiotherapy was started within two weeks of randomisation, using a standardised protocol that relied primarily on daily home exercises as well as 15 visits to an independent physiotherapist (the detailed exercise therapy protocol is available in appendix 1).^13^

#### Diagnostic arthroscopy

**Fig 1:**
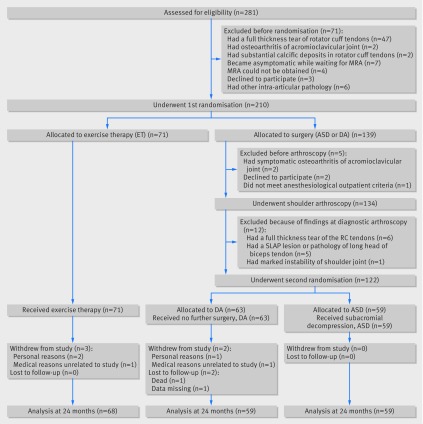
Study flowchart. ASD=arthroscopic subacromial decompression; DA=diagnostic arthroscopy; MRA=magnetic resonance arthrography; SLAP=superior labrum anterior-posterior. Full details of unblinding, treatment conversions, and reoperations are provided in table S7 in appendix 2

We carried out arthroscopic examination of the glenohumeral joint and subacromial space with the use of standard posterior and lateral portals and a 4 mm arthroscope with the patient under general anaesthesia, usually supplemented with an interscalene brachial plexus block. We did an intra-articular and subacromial assessment of the rotator cuff integrity. If the rotator cuff insertion could not be otherwise visualised, subacromial bursal tissue was bluntly stretched with a trochar or resected, keeping the resection to a minimum. If arthroscopic examination showed any pathology needing intervention other than ASD, we excluded the patient from the trial (fig 1). Once the eligibility was confirmed, the participants were randomly assigned to either ASD or diagnostic arthroscopy. For those allocated to the diagnostic arthroscopy group, the operation was terminated. To ensure concealment of the allocation from participants and the staff other than those in the operating theatre, the diagnostic arthroscopy participants were kept in the operating theatre for the time needed to perform subacromial decompression.

#### Arthroscopic subacromial decompression

After arthroscopic examination of the shoulder (that is, diagnostic arthroscopy), the ASD procedure, which involved the debridement of the entire subacromial bursa (bursectomy) and resection of the bony spurs and the projecting anterolateral undersurface of the acromion, was carried out with a shaver, burr, and/or electrocoagulation.^14^

### Postoperative care

The postoperative rehabilitation was identical in the ASD and diagnostic arthroscopy groups, consisting of one visit to an independent physiotherapist, blind to the group assignment, for guidance and instructions for home exercises.

### Outcome measures

Given that the pathognomonic clinical sign of shoulder impingement syndrome is subacromial shoulder pain, especially at night and while lifting the arm, our two primary outcome measures were shoulder pain at rest and shoulder pain on arm activity at 24 months. We used a 0-100 visual analogue scale (VAS) ranging from 0 (no pain) to 100 (extreme pain) to assess the shoulder pain. At the time of our trial’s launch no direct evidence on the appropriate minimal clinically important difference for VAS in patients with shoulder impingement syndrome was available, so we considered 15 points to be the minimal clinically important difference. We based this estimate on an extensive review of the existing literature on minimal clinically important differences for the VAS scale in a wide range of different musculoskeletal conditions. The appropriateness of the chosen minimal clinically important difference has subsequently been validated.^15^

Secondary outcomes included two shoulder function assessment instruments, the Constant-Murley score and the simple shoulder test, as well as the 15D,^16^ a generic health related quality of life instrument made up of 15 dimensions and scored on a scale of 0 (death) to 1 (full health). Patients’ global assessment of satisfaction with the treatment was assessed on a VAS ranging from 0 (completely dissatisfied) to 100 (very satisfied), and satisfaction with the treatment outcome was assessed using a five item scale (from very satisfied to very dissatisfied). We used the responses from the latter question to carry out a responder analysis (appendix 1).

Questionnaires were administered at baseline and three, six, 12, and 24 months after randomisation. The follow-up questionnaires also included a separate section on adverse events. We defined adverse events as untoward medical occurrences that did not necessarily have a causal relation with the treatment administered. Serious adverse events were those having the potential to result in significant disability/incapacity, need inpatient hospital care, prolong the hospital care, be life threatening, or result in death. At the three month follow-up, the surgically treated patients were asked which procedure (ASD or diagnostic arthroscopy) they thought they had had.

### Statistical analysis and sample size calculation

We powered the study to detect a difference of at least the minimal clinically important difference (15 points^15^) in the two primary outcomes between the ASD and diagnostic arthroscopy groups. For the study to have 90% power to show a minimal clinically important advantage of ASD over diagnostic arthroscopy, under the assumption of a two sided type 1 error rate of 5%, we planned to recruit 70 patients per group.

The trial was primarily designed to ascertain whether ASD is superior to diagnostic arthroscopy, at 24 months after the procedure, with the two primary outcomes (the primary confirmatory comparison). We also included a pragmatic comparison of the relative benefits of ASD versus exercise therapy (the secondary exploratory comparison). An independent statistician unaware of the group assignments did all the analyses according to the previously published statistical analysis plan. The statistical analysis plan, outlining our statistical methods in more detail, is provided in appendix 1.

We quantified the treatment effect on an intention to treat (ASD versus diagnostic arthroscopy comparison) or full analysis set (ASD versus exercise therapy comparison) basis, as the difference between the groups in pain scores (VAS), Constant-Murley score, simple shoulder test score, and 15D score with the associated 95% confidence intervals and P values at 24 months after the primary randomisation. In the intention to treat and full analysis set analyses, the participants were included as randomised. We used a mixed model repeated measurements analysis of variance with patient as a random factor (repeated measurements at three, six, 12, and 24 months), the baseline value as a covariate, and assuming a covariance structure with compound symmetry. As the mixed model repeated measurements analysis of variance allows for analysis of unbalanced datasets without imputation, we analysed all available data, the full analysis set. The missingness of the outcome data at different time points is shown in table S12 in appendix 2. We fitted the mixed model repeated measurements model by using the mixed procedure in Stata and used Satterthwaite’s method to calculate the degrees of freedom.

We used generalised estimating equation logistic regression analysis to analyse categorical variables. We compared the frequencies of patients who reported satisfaction or subjective improvement and the proportions of responders and non-responders, those with a change exceeding the minimal clinically important improvement in the primary outcomes, and reoperations/treatment conversions between the two groups at 24 months.

To safeguard against potential multiplicity effects in the primary comparison,^17^ we required a statistically significant treatment effect on both of our primary outcome variables. All secondary analyses are supportive, exploratory, and/or hypothesis generating. We did two sensitivity analyses (per protocol and as treated) and four subgroup analyses (potential effect modifying of the duration and severity of symptoms, the acromial anatomy, and the presence/absence of bursal resection) with the same principles as the intention to treat and full analysis set analyses. We considered a P value of 0.05 to indicate statistical significance. We used Stata v14.1 for all statistical analyses.

### Blinded data interpretation

We interpreted the results of the trial according to a blinded data interpretation scheme.^18^ In brief, an independent statistician provided the Writing Committee of the FIMPACT trial with blinded results from the analyses, with the groups labelled group A, group B, and group C. The Writing Committee then considered the interpretation of the results until a consensus was reached and agreed in writing on all alternative interpretations of the findings. Once a consensus was reached, we recorded the minutes of this meeting in a document coined statement of interpretation, which was signed by all members of the Writing Committee. After this common agreement was reached, the data manager and the independent statistician broke the randomisation code and the correct interpretation was chosen. The draft of the manuscript was then finalised. Detailed minutes of blinded data interpretation meetings are provided in appendix 1.

### Patient involvement

No patients were involved in designing the study, nor were they involved in developing plans for recruitment, design, or implementation of the study. No patients were asked to advise on interpretation or writing up of results. When the results of this randomised controlled trial are published, a lay information flyer with final results will be sent to the recruiting centres for dissemination to the trial participants.

## Results

### Characteristics of patients

Of the 281 eligible patients, we excluded 71 (fig 1). A total of 210 patients were included in the first randomisation; 71 were assigned to exercise therapy and 139 to surgery. Of those allocated to surgery (n=139), another 17 were excluded before the second randomisation (fig 1), leaving 59 patients to receive ASD and 63 to receive diagnostic arthroscopy. Over the course of the 24 month follow-up, three patients in the exercise therapy group and two patients in the diagnostic arthroscopy group withdrew from the study, and one patient in the diagnostic arthroscopy group died. The study groups were well balanced on all baseline characteristics (table 1). The patients who withdrew from the study (n=5) were similar to those who were randomised with respect to primary outcome measures at baseline.

**Table 1:** Baseline characteristics of participants according to study group

Characteristics	Arthroscopic subacromial decompression (n=59)	Diagnostic arthroscopy (n=63)	Exercise therapy (n=71)
Mean (SD) age, years	50.5 (7.3)	50.8 (7.6)	50.4 (6.6)
No (%) female	42 (71)	46 (73)	47 (66)
No (%) dominant hand affected	35 (59)	36 (57)	46 (65)
Mean (SD) duration of symptoms, months	18 (14)	18 (19)	22 (23)
No (%) able to work normally regardless of shoulder symptoms	27 (46)	31 (49)	35 (49)
Mean (SD) visual analogue scale score, at rest*	41.3 (25.8)	41.6 (25.5)	41.7 (27.5)
Mean (SD) visual analogue scale score, on arm activity*	71.2 (23.6)	72.3 (21.7)	72.4 (20.8)
Mean (SD) Constant-Murley score†	32.2 (15.8)	31.7 (14.0)	35.2 (16.2)
Mean (SD) simple shoulder test score‡	4.9 (2.9)	4.9 (2.9)	4.8 (2.7)
Mean (SD) 15D score§	0.89 (0.06)	0.89 (0.07)	0.88 (0.08)

* Shoulder pain at rest and on activity was assessed on a 100 mm visual analogue scale of 0 to 100, with 0 denoting no pain and 100 denoting extreme pain.

† Scoring system for evaluation of various shoulder disorders consisting of both objective (range of motion and strength) and subjective measurements (pain assessment, work load, and leisure time activities), summarised in a score between 0 and 100; higher score indicates better shoulder function.

‡ Based on 12 questions with yes (1) or no (0) response options; maximum score is 12, indicating normal shoulder function; minimum score of 0 points indicates severely diminished shoulder function.

§ Generic health related quality of life instrument comprising 15 dimensions; maximum score is 1 (full health), and minimum score is 0 (death).

### Primary comparison: ASD versus diagnostic arthroscopy

#### Primary outcomes

**Fig 2:**
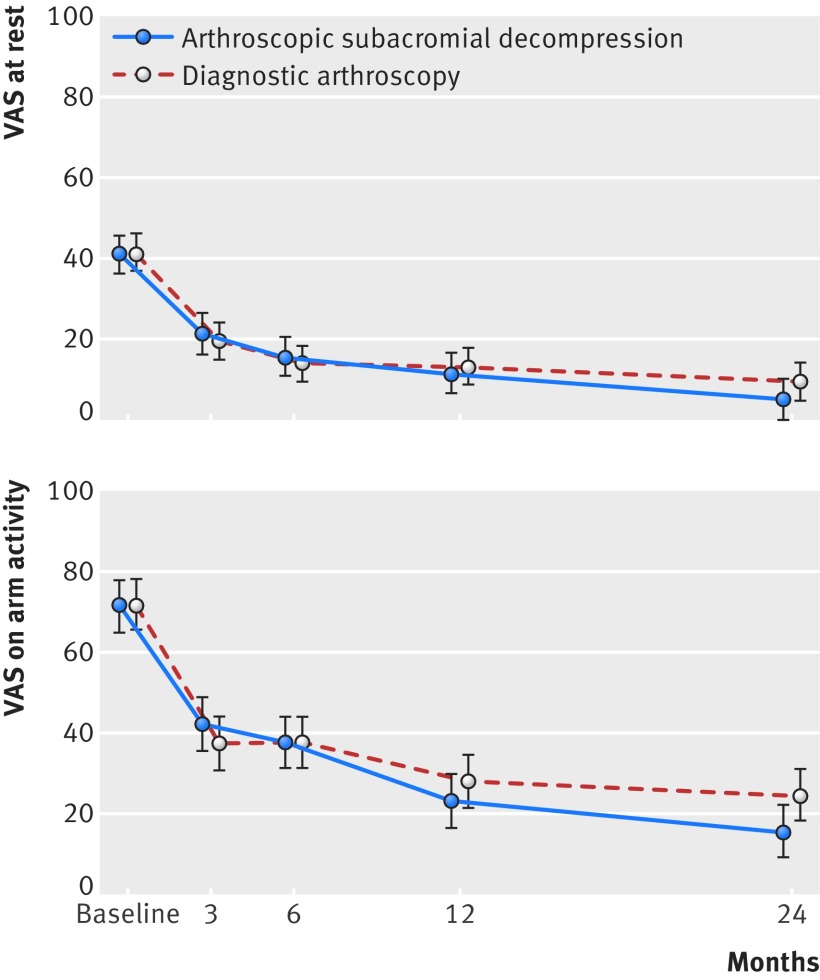
Primary outcomes of primary comparison at baseline and at 3, 6, 12, and 24 month follow-up. Visual analogue scale (VAS) shoulder pain scores at rest and on arm activity over 24 month follow-up period are shown. VAS scales range from 0 to 100, with higher values indicating more severe pain. Data are mean (95% CI) shown at follow-up time points

We saw marked improvement from baseline to 24 months in both primary outcomes in both the ASD and diagnostic arthroscopy groups (mean change for ASD 36.0 at rest and 55.4 on activity; for diagnostic arthroscopy 31.4 at rest and 47.5 on activity) (fig 2 and table 2), but no significant between group differences existed at 24 months in either VAS pain at rest (mean difference, ASD minus diagnostic arthroscopy, −4.6, 95% confidence interval −11.3 to 2.1; P=0.18) or VAS pain on arm activity (−9.0, −18.1 to 0.2; P=0.054) (fig 2, table 2, and table S3 in appendix 2). These results remained unaltered in the pre-specified sensitivity analyses (as treated and per protocol) and subgroup analyses (tables S2, S5, and S6 in appendix 2).

**Table 2:** Primary comparison of arthroscopic subacromial decompression versus diagnostic arthroscopy: outcomes of trial at 24 month follow-up*. Values are mean (95% CI) unless stated otherwise

	Arthroscopic subacromial decompression (ASD; n=59)	Diagnostic arthroscopy (DA; n=59)	Between group difference (ASD *v* DA)†	P value
**Primary outcomes**				
Visual analogue scale score, at rest	5.3 (0.8 to 9.7)	9.9 (5.4 to 14.3)	−4.6 (−11.3 to 2.1)	0.18
Visual analogue scale score, on arm activity	15.8 (9.4 to 22.2)	24.8 (18.4 to 31.2)	−9.0 (−18.1 to 0.2)	0.054
**Secondary outcomes**				
Constant-Murley score	77.9 (73.7 to 82.3)	73.7 (69.5 to 78.0)	4.3 (−2.0 to 10.5)	0.18
Simple shoulder test score	10.3 (9.7 to 10.9)	9.9 (9.3 to 10.5)	0.5 (−0.4 to 1.3)	0.29
15D score	0.92 (0.91 to 0.93)	0.92 (0.91 to 0.93)	0.00 (−0.02 to 0.02)	1.00
Proportion of participants able to return to previous leisure activities‡	0.82 (0.72 to 0.92)	0.77 (0.66 to 0.88)	0.06 (−0.10 to 0.22)	0.45
Proportion of responders§	0.95 (0.89 to 1.0)	0.91 (0.84 to 0.99)	0.04 (−0.06 to 0.14)	0.42
Patients’ satisfaction with treatment¶	88.1 (82.9 to 93.3)	87.1 (81.9 to 92.3)	0.9 (−6.6 to 8.3)	0.82
No (%) complications and adverse effects**	3 (5)	2 (3)	–	–

* Higher score indicates desired (better) treatment outcome for all outcomes other than pain visual analogue scale score and complications, for which lower score indicates better outcomes.

† Between group differences may not exactly equal difference in changes in score between ASD and DA groups because of adjustment for baseline imbalance in mixed effects model.

‡ Ability to return to previous leisure activities was assessed with question “Have you been able to return to your previous leisure activities?” (“yes” or “no”).

§ Participants’ satisfaction with treatment outcome was elicited with question “How satisfied are you with the outcome of your treatment?” on a five item scale; participants who reported being very satisfied or satisfied were categorised as “responders.”

¶ Participants’ global assessment of satisfaction with treatment was elicited with question “Are you satisfied with the treatment you have received?” on visual analogue scale ranging from 0 (completely disappointed) to 100 (very satisfied).

** Complications directly related to interventions were registered.

#### Secondary and other outcomes

We found no significant between group differences in any of the secondary outcomes (table 2 and table S4 in appendix 2). Patients in the diagnostic arthroscopy group were no more likely than those in the ASD group to guess that they had had a placebo procedure (22/53 (42%) and 2154 (39%), respectively; P=0.85).

#### Unblinding of treatment allocation and crossovers

Six of 59 patients in the ASD group and nine of 63 patients in the diagnostic arthroscopy group (P=0.49) reported persistent symptoms after surgery sufficiently severe to lead to unblinding of the study group assignment (at an average of 10 months after the index operation) (table S7 in appendix 2). Two participants in the ASD group underwent a consequent reoperation—one had manipulation under anaesthesia and the other first had acromioclavicular resection and then later manipulation under anaesthesia. In the diagnostic arthroscopy group, eight patients had a reoperation (seven ASDs and one ASD coupled with subscapularis tendon repair). Details of unblindings, treatment conversions, and reoperations are shown in table S7 in appendix 2.

#### Complications and adverse events

One patient in the diagnostic arthroscopy group had temporary swelling in the brachial area related to a brachial plexus block. Three patients in the ASD group and one patient in the diagnostic arthroscopy group developed symptoms consistent with a frozen shoulder over the course of the 24 month follow-up (table 2). No other complications directly related to the interventions were registered.

### Secondary comparison: ASD versus exercise therapy

#### Primary outcomes

**Fig 3:**
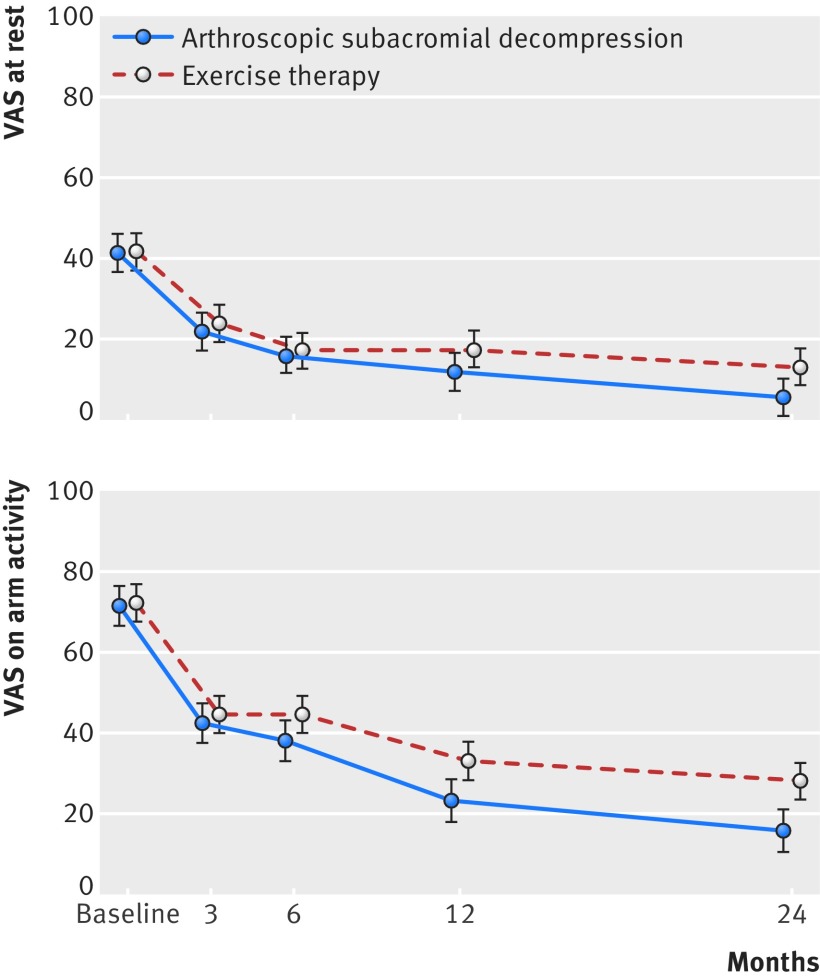
Primary outcomes of secondary comparison at baseline and at 3, 6, 12, and 24 month follow-up. Visual analogue scale (VAS) shoulder pain scores at rest and on arm activity over 24 month follow-up period are shown. VAS scales range from 0 to 100, with higher values indicating more severe pain. Data are mean (95% CI) shown at follow-up time points

Marked improvement from baseline to 24 months was seen in both primary outcomes in both the ASD and exercise therapy groups (fig 3, table 3, and table S8 in appendix 2). We found statistically significant differences in favour of ASD at 24 months in both VAS at rest (−7.5, −14.0 to −1.0; P=0.023) and VAS on arm activity (−12.0, −20.9 to −3.2; P=0.008), but the mean difference between the groups did not exceed the pre-specified minimal clinically important difference of 15. These results remained essentially unaltered in the pre-specified sensitivity analyses (table S10 in appendix 2). We found no significant differences between the groups in the proportion of patients with pain reduction exceeding the minimal clinically important improvement threshold of 15 in VAS pain at rest and VAS pain on activity (table S11 in appendix 2). The proportion of patients with VAS pain on activity below the threshold of 30 at 24 months was lower in the exercise therapy group than in the ASD group (table S11 in appendix 2). Of note, this ASD versus exercise therapy comparison is confounded by lack of blinding and the fact that 17/139 (12%) patients were excluded from the two surgical groups before the second randomisation without any comparable exclusions from the exercise therapy group. As a result, this ASD versus exercise therapy comparison is likely to be biased in favour of ASD. Also, the progressive exercise therapy regimen carried out in the exercise therapy group is different from the overall postoperative care carried out for patients in the ASD group.

**Table 3:** Secondary comparison of arthroscopic subacromial decompression versus diagnostic arthroscopy: outcomes of trial at 24 month follow-up*. Values are mean (95% CI)

	Arthroscopic subacromial decompression (ASD; n=59)	Exercise therapy (ET; n=68)	Between group difference (ASD *v* ET)**†**	P value
**Primary outcomes**				
Visual analogue scale score, at rest	5.3 (0.6 to 10.0)	12.8 (8.4 to 17.3)	−7.5 (−14.0 to −1.0)	0.023
Visual analogue scale score, on arm activity	16.0 (9.6 to 22.5)	28.1 (22.1 to 34.1)	−12.0 (−20.9 to −3.2)	0.008
**Secondary outcomes**				
Constant-Murley score	79.1 (74.7 to 83.4)	71.2 (67.0 to 75.3)	7.7 (1.6 to 13.9)	0.013
Simple shoulder test score	10.3 (9.7 to 10.9)	9.7 (9.1 to 10.2)	0.7 (−0.2 to 1.5)	0.12
15D score	0.91 (0.90 to 0.93)	0.91 (0.90 to 0.92)	0.00 (−0.02 to 0.02)	1.00
Proportion of participants able to return to previous leisure activities‡	0.82 (0.72 to 0.92)	0.76 (0.65 to 0.86)	0.07 (−0.07 to 0.21)	0.31
Proportion of responders§	0.95 (0.90 to 1.01)	0.90 (0.81 to 0.98)	0.06 (−0.04 to 0.16)	0.23
Patients’ satisfaction with treatment¶	88.2 (82.8 to 93.5)	84.9 (79.9 to 89.8)	3.3 (−3.9 to 10.5)	0.36
No (%) complications and adverse effects**	3 (5)	3 (4)	–	–

* Higher score indicates desired (better) treatment outcome for all outcomes other than pain visual analogue scale score and complications, for which lower score indicates better outcomes.

† Between group differences may not exactly equal difference in changes in score between ASD and DA groups because of adjustment for baseline imbalance in mixed effects model.

‡ Ability to return to previous leisure activities was assessed with question “Have you been able to return to your previous leisure activities?” (“yes” or “no”).

§ Participants’ satisfaction with treatment outcome was elicited with question “How satisfied are you with the outcome of your treatment?” on a five item scale; participants who reported being very satisfied or satisfied were categorised as “responders.”

¶ Participants’ global assessment of satisfaction with treatment was elicited with question “Are you satisfied with the treatment you have received?” on visual analogue scale ranging from 0 (completely disappointed) to 100 (very satisfied).

** Complications directly related to interventions were registered.

#### Secondary and other outcomes

The only statistically significant between group difference in the secondary outcomes was in the Constant-Murley score in favour of ASD (7.7, 1.6 to 13.9; P=0.013) (table 3), but the mean difference between the groups did not exceed the pre-specified threshold of 17 for minimal clinically important difference. Furthermore, the group differences in the Constant-Murley score were not statistically significant in the pre-specified sensitivity analyses (table S10 in appendix 2).

#### Unblinding of treatment allocation and crossovers

Fifteen patients who were initially assigned to exercise therapy reported persistent symptoms sufficiently severe to require unblinding; 14 of them subsequently underwent ASD and one underwent acromioclavicular resection. Three consequent reoperations were performed (table S7 in appendix 2).

#### Complications and adverse events

Two patients in the exercise therapy group developed symptoms consistent with a frozen shoulder, and one patient reported aggravation of low back pain over the course of exercise therapy regimen (table 3). No other adverse events directly related to the exercise therapy were registered.

## Discussion

This multicentre, randomised, placebo controlled trial involving patients with shoulder impingement syndrome showed that arthroscopic subacromial decompression was not superior to diagnostic arthroscopy, with regard to outcomes assessed at the end of a 24 month follow-up period. Although both groups had significant improvement in both primary outcomes, the patients assigned to ASD had no clinically relevant improvement over those assigned to diagnostic arthroscopy.

### Comparison with other studies

We are aware of only one other randomised, placebo surgery controlled trial on the efficacy of ASD in the treatment of shoulder impingement syndrome.^19^ The findings of this recently published “Can Shoulder Arthroscopy Work?” (CSAW) trial showed that at both the primary six month follow-up and the secondary 12 month follow-up, arthroscopic subacromial decompression seemed to offer no extra benefit over arthroscopy alone (placebo comparator).^20^ The findings are in full agreement with the short term (six and 12 month) findings of our trial (fig 2 and table S3 in appendix 2) with respect to efficacy of ASD over diagnostic arthroscopy. Our trial further shows no clinically relevant benefit of ASD over diagnostic arthroscopy at our primary, 24 month, follow-up time point.

### Strengths and limitations of study

The placebo surgery controlled design represents the primary difference between our trial and the CSAW trial^20^ and the rest of the existing literature on this topic. Acknowledging that the act of surgery in itself produces a profound placebo response,^21^ ^22^ ^23^ the actual treatment effect is impossible to distinguish from the nonspecific (and placebo) effects—such as the patients’ or researchers’ expectations of benefit—without a placebo comparison group.^24^ Such bias is particularly important in trials with subjective endpoints.^25^ Given that the proportions of patients who guessed whether they had undergone a placebo procedure was similar in the two surgical groups, we argue that the risk of performance bias is low in our trial. Diagnostic arthroscopy controlled trials in the knee and shoulder with a very similar design to our study have prompted assertions that diagnostic arthroscopy cannot be considered a true placebo comparator because of the alleged therapeutic effects of joint lavage.^26^ ^27^ ^28^ ^29^ The existing high quality evidence disputes such assertions, as tidal irrigation and arthroscopic lavage have both failed to provide a benefit over placebo procedures (placebo irrigation or skin incisions, respectively).^22^ ^30^ Moreover, no concerns were expressed regarding the validity of using diagnostic arthroscopy as a control in a previous placebo surgery controlled trial on surgery after shoulder dislocation.^31^ One obvious advantage of appropriate blinding became readily apparent in our trial. In the previous (unblinded) trials comparing ASD with conservative treatment alternatives,^13^ ^32^ ^33^ ^34^ the observed higher frequency of crossovers in the conservatively treated patients has been interpreted as evidence for the superiority of ASD over conservative treatment. However, we note that the decision to (re)operate was made after unblinding of the treatment group allocation, whereas the decision to unblind the treatment group is made without awareness of the treatment given to the patient. We thus consider the frequency of “unblindings” a less biased measure of the severity of participants’ symptoms than the frequency crossovers.^35^ In our trial, we found no statistically significant difference in the frequency of unblindings between the ASD and diagnostic arthroscopy groups (6/59 in the ASD group versus 9/63 in the diagnostic arthroscopy group; P=0.49).

Besides the placebo control, another obvious strength of the ASD versus diagnostic arthroscopy comparison was the efficacy or mechanistic design.^36^ We used highly experienced surgeons and therapists and isolated the critical therapeutic element of the ASD procedure—the subacromial decompression—as the only difference between the two surgical groups while carefully maintaining all other care as close to identical as possible. In particular, we used very stringent eligibility criteria to enrol—according to best available evidence—only patients most likely to benefit from ASD. Classically, stringent eligibility criteria are considered to decrease the external validity of a study. Although our patient population was indeed highly selected, as showcased by the lengthy recruitment period needed despite three high volume centres, we think that the use of stringent eligibility criteria paradoxically increases the generalisability of our findings. When ASD was proven futile under this best case scenario, there is no reason to assume that it would work better under less optimal circumstances or in a more heterogeneous population. We also note that our primary findings are robust, as the sensitivity and subgroup analyses did not materially change the findings of our primary analyses (tables S5 and S6 in appendix 2). In essence, the duration or severity of symptoms or the acromial anatomy—factors previously asserted as potential modifiers of the effect of ASD—did not have a hypothesised effect on the primary outcomes (table S6 in appendix 2). Obviously, all our subgroup analyses are at higher risk of bias and should be considered only supportive, explanatory, and/or hypothesis generating.

Some limitations warrant discussion. The number of participants completing the entire two year follow-up was 59 in both the ASD group and the diagnostic arthroscopy group, below the pre-specified target of 68. The high number of exclusions among the participants allocated to surgery was primarily attributable to unexpectedly poor sensitivity of magnetic resonance arthrography in detecting rotator cuff tears and other pathology needing intervention other than ASD. Although our sample size being below the pre-specified target might prompt assertions that the study is underpowered, we note that our point estimates exclude clinically significant treatment effects. In essence, our findings are not based on absence of evidence, as in an underpowered study, but rather on evidence of absence of a clinically significant treatment benefit. One may also criticise the validity of the chosen minimal clinically important difference threshold. At the time of designing the trial, no evidence existed on the appropriate minimal clinically important difference for patients with shoulder impingement syndrome, so instead of being based on empirical data our estimate for the minimal clinically important difference (15 VAS points) was based on extensive review of the literature in a wide range of different musculoskeletal conditions. Reassuringly, some years after the launch of our trial, a study exploring the minimal clinically important difference for the pain VAS in rotator cuff disease reported a point estimate of 14 VAS points.^15^

Some evidence also suggests that bursectomy alone (complete resection of the subacromial bursa) provides similar outcomes to subacromial decompression (bursectomy accompanied by resection of acromial bone) in patients with shoulder impingement syndrome.^37^ ^38^ ^39^ Acknowledging this, while also appreciating that a rotator cuff tendon tear is considered a different clinical entity from shoulder impingement and a potentially strong prognostic factor for poor outcome, we were faced with a methodological dilemma between the elimination of the presence of a clinically relevant rotator cuff tear versus possible confounding caused by a need to carry minimal resection of the subacromial bursal tissue to properly visualise rotator cuff tendon insertion. We chose to prioritise the rotator cuff tears; accordingly, bursa was either bluntly stretched with trochar or resected if adequate visualisation of the rotator cuff insertion could not be achieved otherwise. Bursal resection was carried out in 18 (30%) of the 63 participants in the diagnostic arthroscopy group; in all but three cases, the resection was minimal. To assess the possible effect of this bursal tissue resection on our findings, we did a pre-specified post hoc analysis (table S2 in appendix 2). Although underpowered, the analysis did not show any statistically significant differences in the primary outcomes between patients who had resection carried out and those who did not. If anything, the observed marginal differences favoured no resection.

Furthermore, on the decision to carry out bursal resection, despite our thorough preoperative screening that included both careful clinical examination and magnetic resonance imaging with contrast, roughly 4% (6/134) of patients having shoulder arthroscopy had to be excluded owing to a rotator cuff tear found at arthroscopic examination. Conventional wisdom dictates that the preferred treatment for rotator cuff tears is to repair partial thickness tears that involve more than 50% of the tendon thickness (grade III), whereas those that involve less than 50% of the tendon thickness (grades I and II) can be treated with debridement, with or without accompanying subacromial decompression.^40^ In this trial, we chose to adhere to this treatment strategy, although its veracity—the need to repair grade III/full thickness rotator cuff tears of degenerative origin—can be questioned according to the most recent high quality evidence.^41^ Finally, a frozen shoulder is considered a potential complication of the treatment of patients with shoulder impingement syndrome, particularly of shoulder arthroscopy.^42^ However, at the early stages of the disease, the clinical presentation of a slowly developing frozen shoulder can mimic subacromial impingement, so a legitimate concern exists that some of the participants we labelled as having developed a frozen shoulder as a complication of treatment might actually initially have been misdiagnosed as having shoulder impingement syndrome while actually having a frozen shoulder in the first place. In the end, the number of patients labelled having developed a frozen shoulder was small in all groups (two, three, and one in the exercise therapy, ASD, and diagnostic arthroscopy groups, respectively).

In addition to our primary sham surgery controlled efficacy comparison between ASD and diagnostic arthroscopy, our study also included a pragmatic, exploratory secondary comparison between surgical and non-operative care (ASD versus exercise therapy). In apparent contrast to four previous, similar randomised trials that found no benefit of ASD over various exercise therapy regimens,^13^ ^32^ ^33^ ^34^ we observed a statistically significant benefit of ASD over exercise therapy in both our primary outcomes. Although the benefit did not exceed the pre-specified minimal clinically important difference (15 point change in VAS) in either of the two primary outcomes (table 3), a potential beneficial effect of ASD over exercise therapy cannot be completely ruled out, as the confidence intervals for the mean difference in pain VAS on arm activity include the minimal clinically important difference. In interpreting the findings for ASD versus exercise therapy, one needs to appreciate several concerns related to this comparison. Firstly, this is not a blinded comparison as the participants are naturally aware of the treatments given and thus the results are inevitably confounded by potentially different placebo effects related to the surgical and nonoperative care. Secondly, a clear prognostic imbalance exists between the two interventions owing to the exclusions carried out before the second randomisation in the group primarily allocated to surgery: 17 (12%) of the 139 participants allocated to the two surgical groups were excluded without any comparable exclusions from the exercise therapy group. Thus, the ASD versus exercise therapy comparison is likely to be biased in favour of ASD owing to the systematic removal of patients with likely poorer prognosis. Finally, the ASD and exercise therapy groups cannot be considered fully comparable owing to differences in the treatment given. The progressive exercise therapy regimen carried out in the exercise therapy group is different from the postoperative rehabilitation carried out by patients in the ASD group, as surgically treated patients need time to recover from the initial surgical trauma while also being subject to some degree of postoperative immobilisation, extended sick leave, and modifications in pain medication and activities. In summary, the results of our secondary comparison (ASD versus exercise therapy) should be interpreted with caution, as we do not know whether exercise therapy is poorer because of the lack of comparability of the groups, because exercise therapy is truly a less effective treatment, or a mixture of both.

### Conclusions and policy implications

The results of this randomised, placebo surgery controlled trial show that arthroscopic subacromial decompression provides no clinically relevant benefit over diagnostic arthroscopy in patients with shoulder impingement syndrome. The findings do not support the current practice of performing subacromial decompression in patients with shoulder impingement syndrome.

**What is already known on this topic**

Arthroscopic subacromial decompression, the most commonly performed shoulder surgery, is carried out to treat patients with shoulder impingement syndrome
Three recent systematic reviews indicate that subacromial decompression is not superior to exercise therapy in patients with shoulder impingement syndrome
Without a placebo surgical comparator (proper blinding), the efficacy of arthroscopic subacromial decompression cannot be assessed

**What this study adds**

This FIMPACT trial and the recently published (highly similar) CSAW trial are the first two placebo surgery controlled trials on the efficacy of arthroscopic subacromial decompression
Both arthroscopic subacromial decompression and diagnostic arthroscopy (placebo surgery) resulted in significant improvements in pain and functional outcomes with no difference in the incidence of adverse events
However, the patients assigned to arthroscopic subacromial decompression had no superior improvement over those assigned to diagnostic arthroscopy

## Web Extra

Extra material supplied by the author

## Notes

Contributors: The corresponding author attests that all listed authors meet authorship criteria and that no others meeting the criteria have been omitted. MP and TJ were co-principal investigators. MP, AM, ST, and TJ conceived and designed the study, and JI also contributed to the design. KK, JK, IS, and VS recruited most of the patients, FIMPACT Investigators followed up the patients. MP, AM, ST, KK, and TJ collected the data. ST designed the database and cleaned the data. ST, TJ, and JR contributed to the study analysis plan and data analysis. MP, ST, KK, AM, JR, and TJ participated in the analysis and interpretation of the data, JR led the blinded data analysis process. TJ, ST, KK, MP, JR, and AM drafted the manuscript. All authors contributed to final data interpretation and contributed to and approved the final draft of the manuscript. TJ obtained funding. MP is the guarantor.

Steering Committee: MP, TJ, AM, and ST.

Writing Committee: TJ, ST, MP, KK, and AM.

Participating investigators (FIMPACT Investigators): Mika Paavola, Teppo Järvinen, Simo Taimela, Antti Malmivaara, Kari Kanto.

Participating clinical sites: Helsinki University Hospital, Jorvi Hospital: Kalevi Hietaniemi, Juha Kalske, Vesa Lepola, Jyrki Salmenkivi, Sikri Tukiainen; Helsinki University Hospital, Herttoniemi Hospital: Jarkko Pajarinen, Mikko Salmela, Vesa Savolainen, Ilkka Sinisaari; Hatanpää City Hospital, Tampere: Timo Järvelä, Kari Kanto, Janne Lehtinen, Mikael Salmela.

FIMPACT Methods Centre: Leena Caravitis, Sari Karesvuori, Pirjo Toivonen (project management), Mathias Bäck (data management), Ville Haapamäki (imaging), Jari Inkinen (physiotherapy), Esa Läärä (randomisation), Harri Sintonen (health related quality of life outcomes).

Ethical approval: This study was approved by the Institutional Review Board of the Pirkanmaa Hospital District (R04200; 28 December 2004), and informed consent was obtained from all patients.

Data sharing: FIMPACT data are not publicly available owing to data privacy issues, but access to the anonymised dataset can be obtained from the corresponding author on reasonable request.

Transparency: The lead author affirms that this manuscript is an honest, accurate, and transparent account of the study being reported; that no important aspects of the study have been omitted; and that any discrepancies from the study as planned (and, if relevant, registered) have been explained.
