# Supplementary material for: Subacromial decompression versus diagnostic arthroscopy for shoulder impingement: randomised, placebo surgery controlled clinical trial
Source: BMJ. 2018 Jul 19;362:k2860. doi: 10.1136/bmj.k2860 (PMC6052435; doi:10.1136/bmj.k2860)
Supplement: Supplementary file 1 — Appendix 1 [file paam040473.ww1.pdf]

Supplementary appendix 1

Table of Contents

Exercise Therapy (ET) Protocol 2
Patient Satisfaction and the Responder Analysis 19
Statistical Analysis Plan (SAP) 20
Minutes of the Blinded Data Interpretation Meeting 29

This supplementary material has been provided by the authors to give readers additional information about their work.

Exercise Therapy (ET) Protocol

**Phase I (weeks 0-3)**	
**Goals: Decrease pain, restore A/PROM, relieve pain and inflammation, re-establish muscular balance, and improve posture; Inform the patient**	
**Passive exercises:**<br>PROM<br>Joint mobilisation/posterior capsule stretching<br>Soft tissue mobilisation	**Active exercises:**<br>AAROM<br>Submaximal isometric rotator cuff exercises<br>Thoracic spine mobilisation<br>Scapular retraction/protraction
**Phase II (weeks 4-5)**	
**Goals: Re-establish full and pain free AROM, restore rotator cuff strength, restore normal scapulothoracic motion**	
**Passive exercises:**<br>Joint mobilisation/posterior capsule stretching<br>Soft tissue mobilisation	**Active exercises:**<br>AROM<br>Maximal isometric rotator cuff exercises<br>Thoracic spine mobilisation<br>Scapulothoracic motion
**Phase III (weeks 6-8)**	
**Goals: Restore muscle strength and endurance, re-establish full and pain free AROM. Restore normal scapulothoracic motion**	
**Passive exercises:**<br>Joint mobilisation	**Active exercises:**<br>Dynamic rotator cuff exercises<br>Scapulothoracic motion
**Phase IV (weeks 9-12)**	
**Goals: Enhance muscle strength and endurance, re-educate neuromuscular control of rotator cuff muscles**	
**Passive exercises:**<br>Continued stretching program	**Active exercises:**<br>Continued dynamic rotator cuff program

Abbreviations: PROM, Passive range of motion; AAROM, Active-assisted range of motion; AROM: Active range of motion

Exercise therapy (ET) group: home exercise program

Phase I (0-3 weeks)

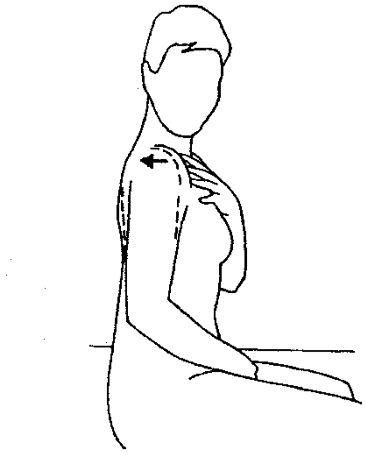

© PhysioTools Ltd

Sit. Place your hand on the front of the opposite shoulder. Feel your shoulder with your fingers making sure that the shoulder does not come forward. Move your shoulder gently 1 cm back and 2 cm up. Later on, exercise is done without palpation or visual confirmation of movement.

Repeat 3 x 15-25 times.

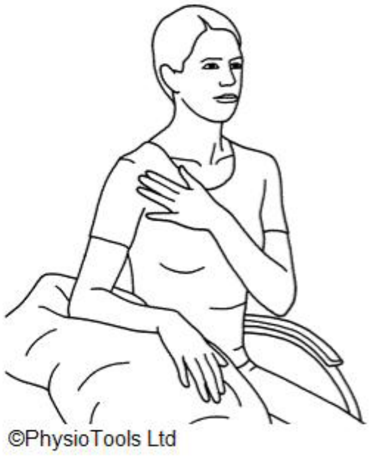

Sit. Place your hand on the front of the opposite shoulder. Feel your shoulder with your fingers. Move your shoulder gently 1 cm forward and 2 up from resting position. Later on, exercise is done without palpation or visual confirmation of movement.

Repeat 3 x 15-25 times.

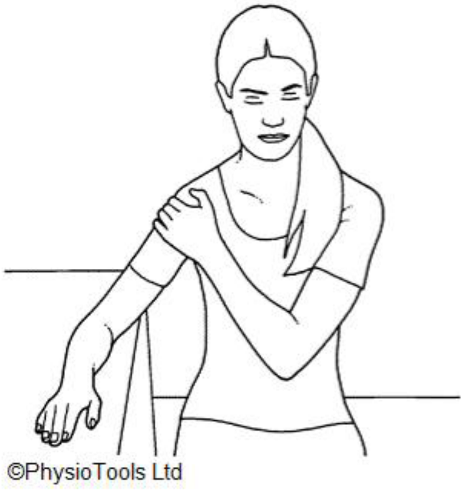

Sit on a chair with your arm supported on a table.
With your other hand push the top of your upper arm downwards.
Hold approx. 2 secs

Repeat 10 times.

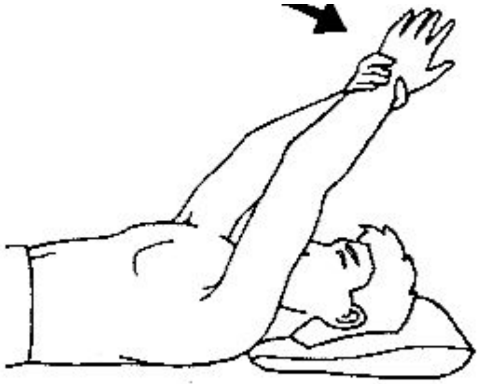

© PhysioTools Ltd

Lying on your back with elbows straight. Use one arm to lift the other arm up keeping it as close to the ear as possible.

Repeat 10 times.

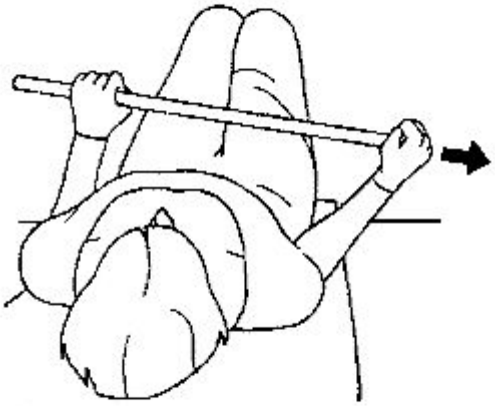

© PhysioTools Ltd

Lying on your back with elbows against your body and at a right angle. Hold a stick in your hands. Move the stick sideways thus pushing the arm to be exercised outwards.

Repeat 10 times.

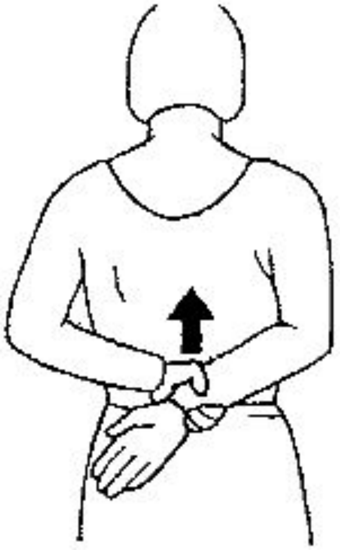

© PhysioTools Ltd

Stand with arms behind your back. Grasp the wrist of the arm you want to exercise. Slide your hands up the back.

Repeat 10 times.

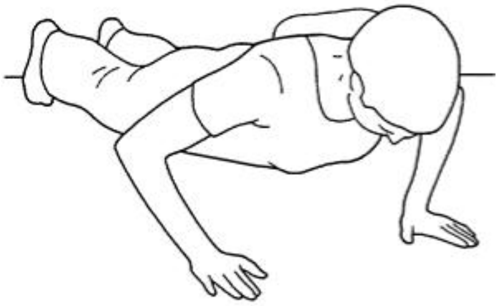

©PhysioTools Ltd

Lying face down with your hands on the floor at shoulder height. Do push-ups slowly and remember to straighten your elbows properly.

Repeat 3 x 15-25 times.

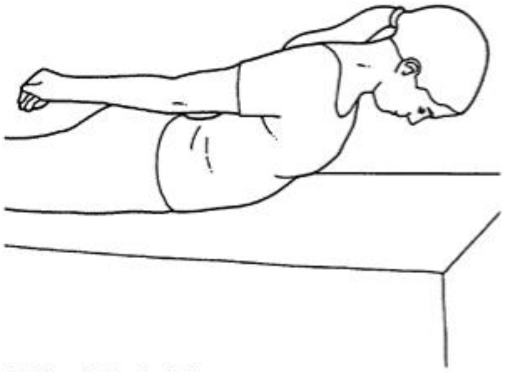

©PhysioTools Ltd

Lying face down, arms behind your back. Lift your upper trunk off the floor and pull your shoulder blades together. Look down at the floor while doing the exercise.

Repeat 3 x 15-25 times.

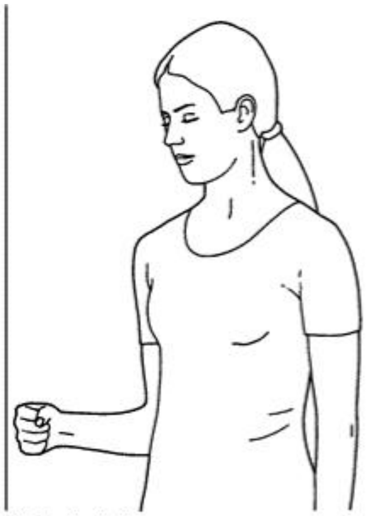

©PhysioTools Ltd

Stand sideways against a wall with your upper arm close to your side and elbow at a right angle. Push the forearm to the side against the wall. Hold approx. 5 secs.

Repeat 3 x 3 times.

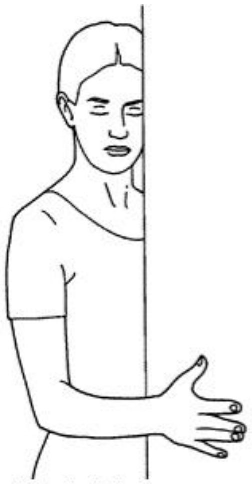

©PhysioTools Ltd

Stand in a doorway with you elbow close to your body and bent at a right angle. Place your hand against the wall. Push your hand inwards against the wall. Hold 5 secs. Relax.

Repeat 3 x 3 times.

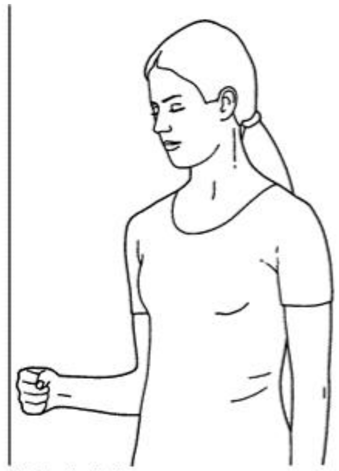

©PhysioTools Ltd

Stand with your upper arm close to your side, elbow at a right angle and the back of your hand against a wall. Push the back of your hand against the wall. Hold approx. 5secs.

Repeat 3 x 3times.

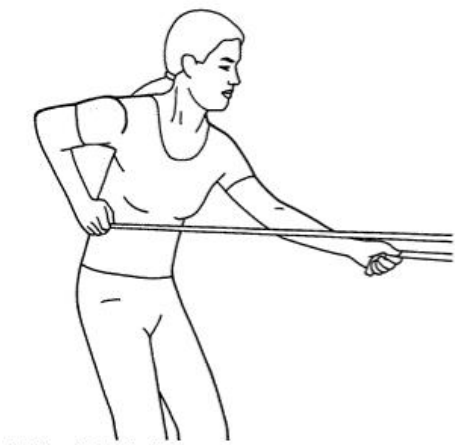

©PhysioTools Ltd

Stand facing a rubber exercise band with your knees and hips slightly bent. Pull the band alternately with the left and right hand keeping the pelvis still.

Repeat 3 x 15-25 times.

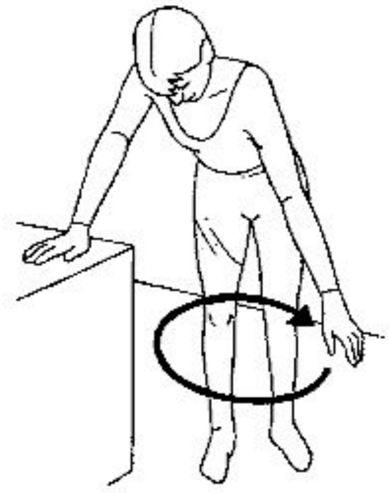

© PhysioTools Ltd

Stand leaning on a table with one hand. Let your other arm hang relaxed straight down. Swing your arm as if drawing a circle on the floor. Change direction.

Continue for 1-2 minutes

Phase II (4-5 weeks)

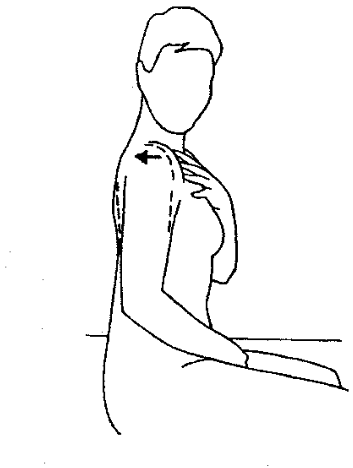

© PhysioTools Ltd

Sit. Place your hand on the front of the opposite shoulder. Feel your shoulder with your fingers making sure that the shoulder does not come forward. Move your shoulder gently 1 cm back and 2 cm up. Later on, exercise is done without palpation or visual confirmation of movement.

Repeat 3 x 15-25 times.

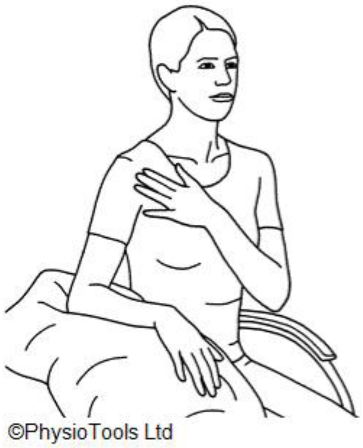

Sit. Place your hand on the front of the opposite shoulder. Feel your shoulder with your fingers. Move your shoulder gently 1 cm forward and 2 up from resting position. Later on, exercise is done without palpation or visual confirmation of movement.

Repeat 3 x 15-25 times.

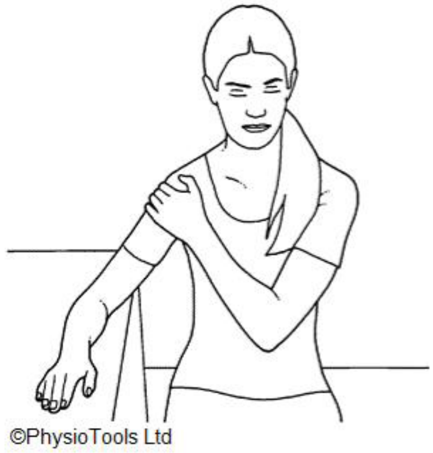

Sit on a chair with your arm supported on a table.
With your other hand push the top of your upper arm downwards. Hold approx. 2 secs.

Repeat 10 times.

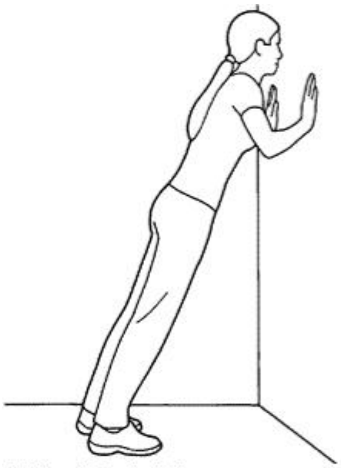
©PhysioTools Ltd

Stand facing a wall with your arms straight and hands on the wall. Do push-ups against the wall keeping your body in a straight line.

Repeat 3 x 15-25 times.

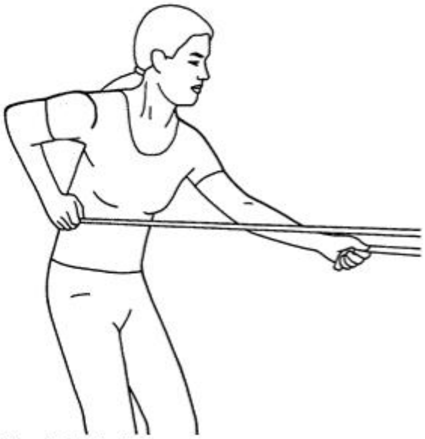
©PhysioTools Ltd

Stand facing a rubber exercise band with your knees and hips slightly bent. Pull the band alternately with the left and right hand keeping the pelvis still.

Repeat 3 x 15-25 times.

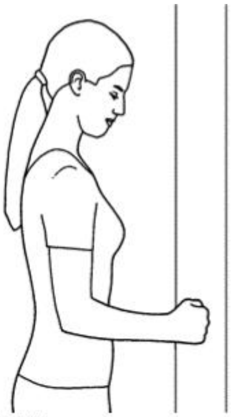
©PhysioTools Ltd

Stand facing a wall. Keep your upper arm close to the side with elbow at a right angle. Push your fist against the wall for 5 secs.

Repeat 3 x 3 times.

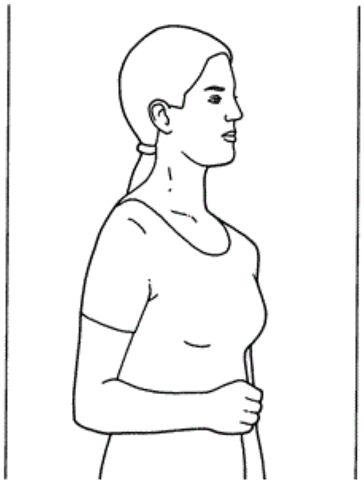
©PhysioTools Ltd

Stand with your back against the wall. Keep your upper arm close to the side and elbow at a right angle. Push the elbow back against the wall and hold for 5 secs.

Repeat 3 x 3 times.

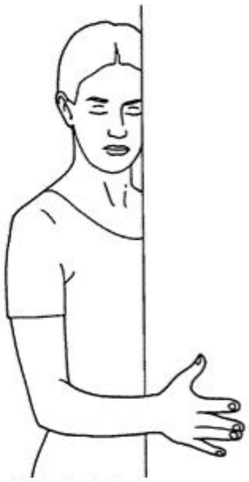
©PhysioTools Ltd

Stand in a doorway with you elbow close to your body and bent at a right angle. Place your hand against the wall. Push your hand inwards against the wall. Hold 5 secs.

Repeat 3 x 3 times.

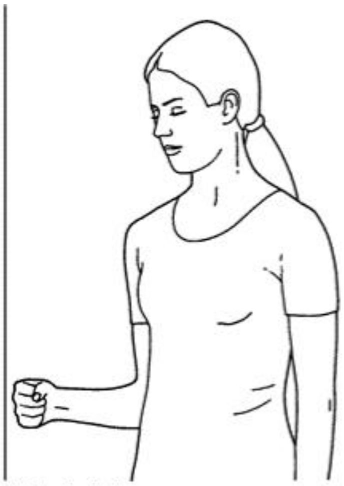
©PhysioTools Ltd

Stand with your upper arm close to your side, elbow at a right angle and the back of your hand against a wall. Push the back of your hand against the wall. Hold approx. 5 secs.

Repeat 3 x 3times.

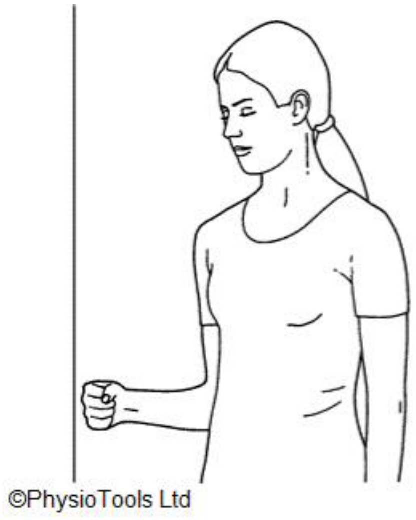

Stand sideways against a wall with your upper arm close to your side and elbow at a right angle. Push the forearm to the side against the wall. Hold approx. 5 secs.

Repeat 3 x 3 times.

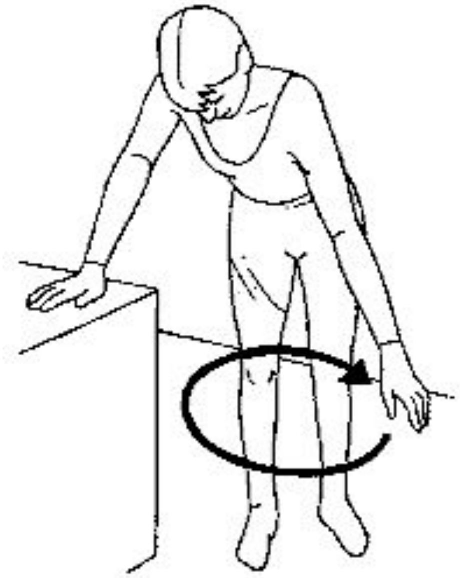

© PhysioTools Ltd

Stand leaning on a table with one hand. Let your other arm hang relaxed straight down. Swing your arm as if drawing a circle on the floor. Change direction.

Continue for 1-2 minutes

Phase III (6-8 weeks)

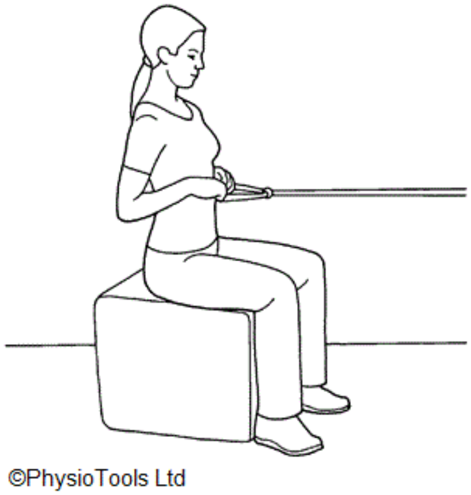

Sit or stand holding on to a rubber exercise band with both hands. Pull the band with both arms pushing the shoulder blades together.

Repeat 3 x 15-25 times.

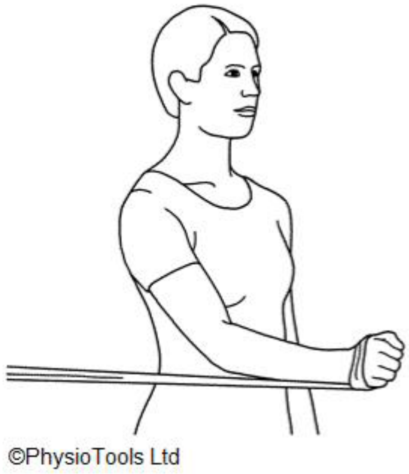

Stand straight holding an exercise band. Move your arm forward, pulling the band. Slowly return.

Repeat 3 x 15-25 times.

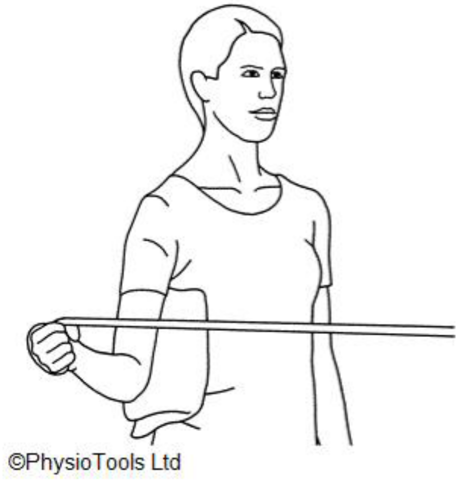

Stand or sit. Place a rolled towel between your upper arm and side. Your upper arm should be slightly forward and your elbow at a right angle. Hold an exercise band. Pull the band taking your forearm out 45 degrees. Hold for 5 secs.

Repeat 3 x 15-25 times.

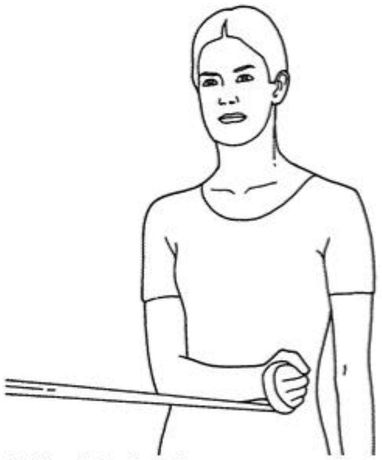

©PhysioTools Ltd

Stand keeping your upper arm close to the side and elbow at a right angle. Hold a rubber exercise band. Pull the band toward your stomach and hold for 5 secs.

Repeat 3 x 15-25 times.

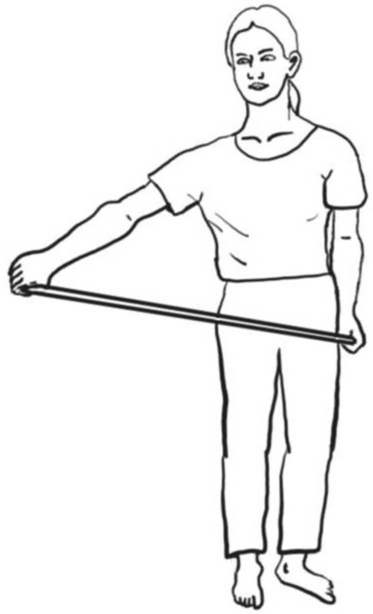

Hold an exercise band with both hands. Pull the band outwards to about 45-60 degrees with back of your hand leading the movement. Hold for 5 secs.

Repeat 3 x 15-25 times

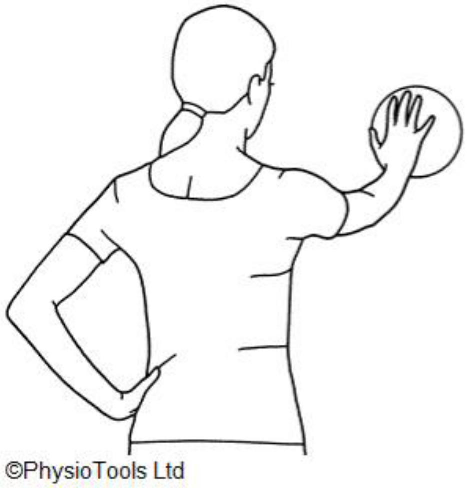

©PhysioTools Ltd

Stand with one foot forward. Keep your back straight. Place the palm of your hand against a wall using a ball or a piece of cloth. Lean your body weight onto your hand. Control your shoulder position and move hand in small horisontal motions.

Repeat 3 x 15-25 times.

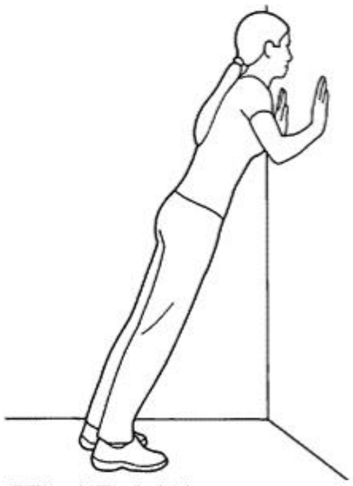

Stand facing a wall with your arms straight and hands on the wall. Do push-ups against the wall keeping your body in a straight line.

Repeat 3 x 15-25 times.

Phase IV (9-12 weeks)

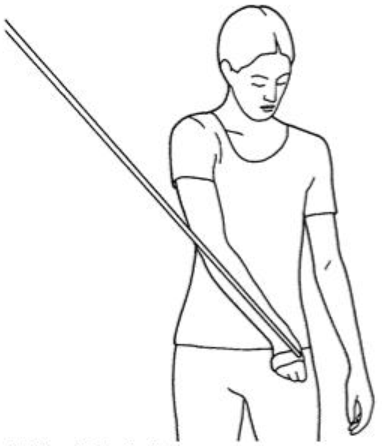

©PhysioTools Ltd

Stand with your arm up and out to the side. Hold a rubber exercise band. Pull the band down and across your body letting your thumb lead the movement.

Repeat 3 x 15-25 times.

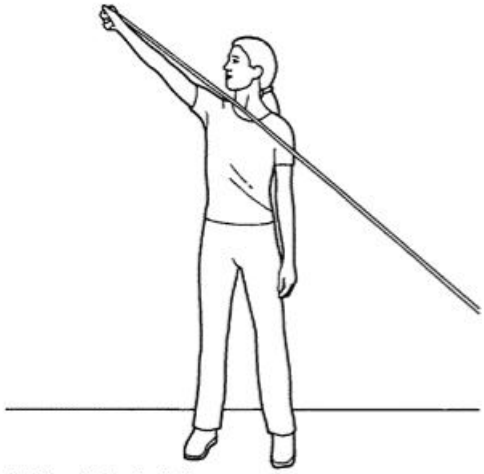

©PhysioTools Ltd

Stand or sit with the hand of the arm to be exercised on your opposite hip. Hold on to a rubber exercise band. Pull the band up towards the opposite side.

Repeat 3 x 15-25 times.

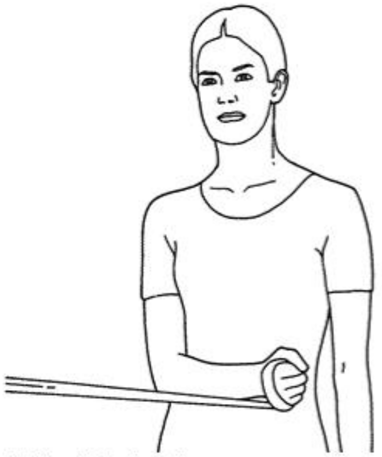

©PhysioTools Ltd

Stand keeping your upper arm close to the side and elbow at a right angle. Hold a rubber exercise band. Pull the band toward your stomach.

Repeat 3 x 15-25 times.

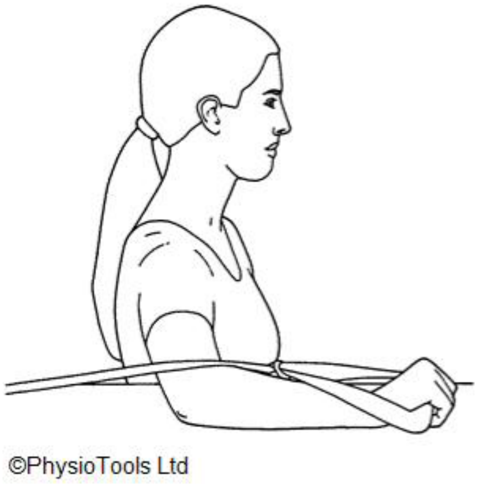

Alternatively,
Sit on a chair with your arm lifted to the side and your elbow at a right angle supported on a table. Hold on to a rubber exercise band which is fastened behind you. Pull the band keeping elbow bent and resting on the table.

Repeat 3 x 15-25 times.

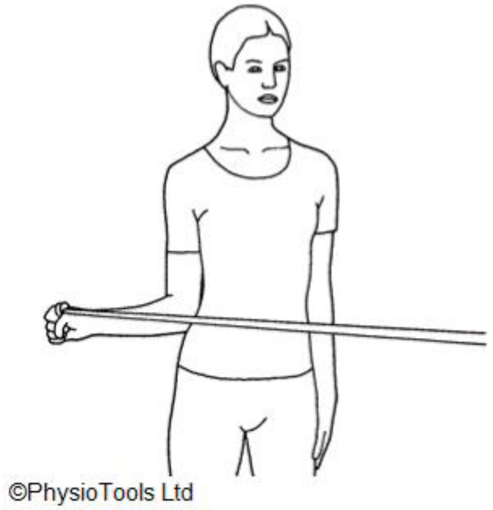

Stand keeping your upper arm close to the side and elbow at a right angle. Hold a rubber exercise band. Pull the band by turning your forearm outwards.

Repeat 3 x 15-25 times.

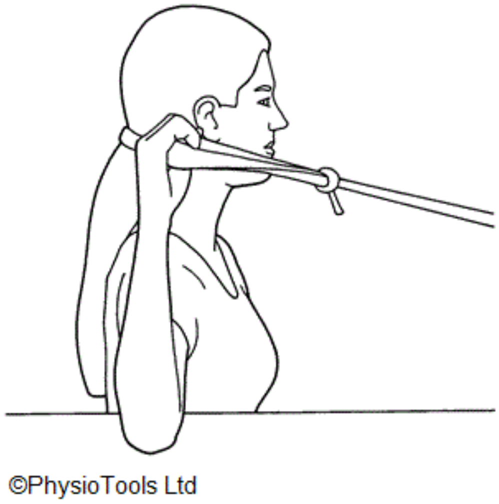

Alternatively,
Sit on a chair with your arm lifted out to the side and elbow at a right angle supported on a table. Hold on to a rubber exercise band which is in front on you. Pull the band keeping your elbow bent and resting on the table.

Repeat 3 x 15-25 times.

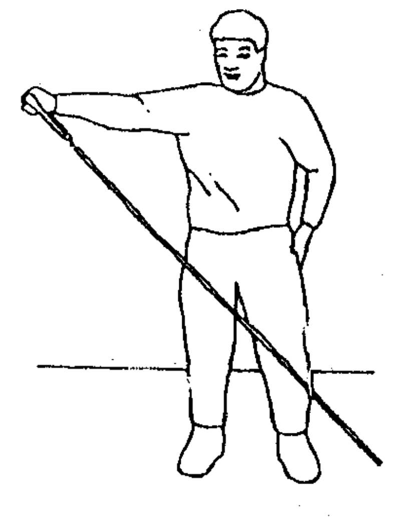

Stand or sit with the hand of the arm to be exercised at your side. Hold on to a rubber exercise band attached at floor level. Pull the band up upwards up to 90 degrees.

Repeat 3 x 15-25 times.

© PhysioTools Ltd

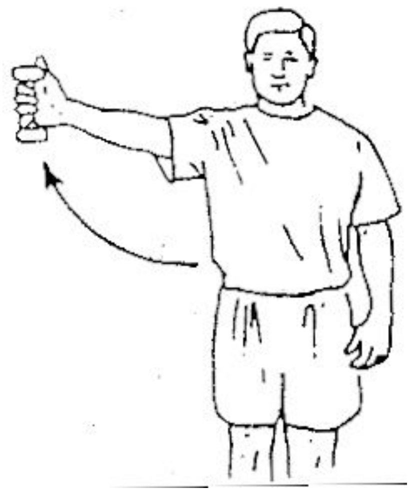

Stand or sit with the hand of the arm to be exercised at your side. Lift hand in thump up position up to a 90° of abduction.

Repeat 3 x 15-25 times.

© PhysioTools Ltd

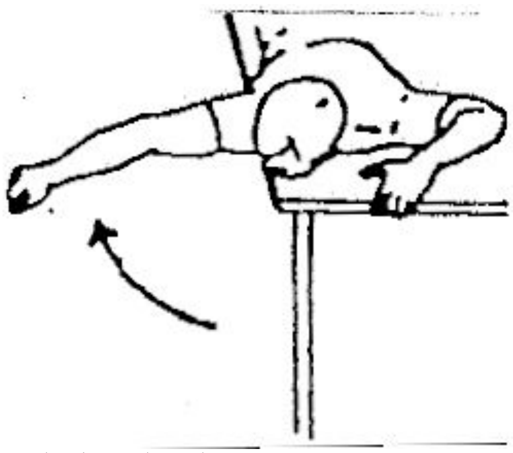

Lying face down with your arms out to the side. Lift your arm toward the ceiling with back of your hand leading the movement.

Repeat 3 x 15-25 times.

© PhysioTools Ltd

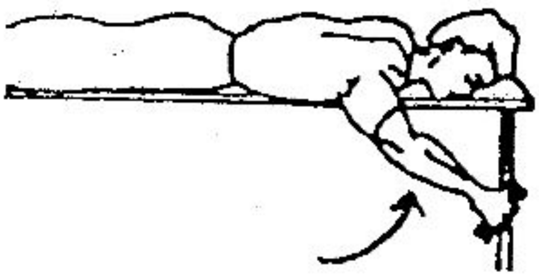

© PhysioTools Ltd

Lying face down with your arms out to the side. Lift your arm forwards up to 100° of flexion with your thumbs leading the movement.

Repeat 3 x 15-25 times.

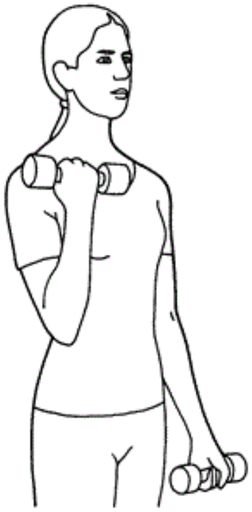

©PhysioTools Ltd

Stand with arms hanging down. Hold a hand weights and turn your palms forward. Bend alternate elbows briskly.

Repeat 3 x 15-25 times.

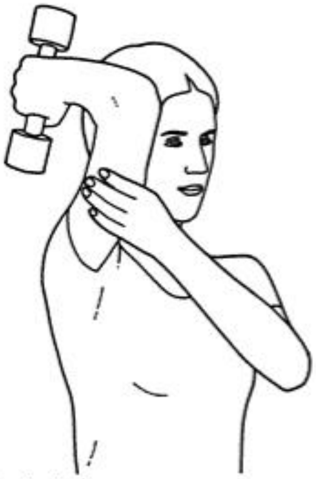

©PhysioTools Ltd

Sit or stand holding a kg hand weight. Bring the arm to be exercised up with the elbow pointing to the ceiling. Support the elbow with the other hand. Straighten the arm holding the weight.

Repeat 3 x 15-25 times.

Patient Satisfaction and the Responder Analysis

Global assessment of satisfaction with the treatment

How satisfied are you with the treatment given? Place a vertical mark to the line below to indicate your current satisfaction to the treatment given.

Completely dissatisfied ____Very satisfied

Satisfaction with the treatment outcome

How satisfied are you with the treatment outcome? Mark the answer closest to your situation.

1. Very satisfied, my shoulder has healed completely.
2. Satisfied, I have only minor, activity related symptoms. My shoulder is much better than before treatment.
3. Somewhat satisfied, I have only minor symptoms. My shoulder is better than before treatment.
4. Dissatisfied, my shoulder is the same as before treatment.
5. Very dissatisfied, my shoulder is worse than before treatment

Responder analysis

Instead of focusing only on the statistical significance of the mean differences between treatment groups in the VAS (i.e., the mean improvement from baseline to 24 months), "a responder analysis" was also carried out. We anchored our responder analysis to the patient's assessment of satisfaction with the shoulder treatment outcome: Patients reporting very satisfied or satisfied were categorised as "Responders".

Statistical Analysis Plan (SAP)

Finnish Subacromial Impingement Arthroscopy Controlled Trial (FIMPACT), 2-year follow-up

Simo Taimela[1] and Teppo L N Järvinen[1]

Statistical advisor:

Professor Jonas Ranstam (Lund, Sweden)

[1] Department of Orthopedics and Traumatology, Helsinki University Central Hospital and University of Helsinki, Helsinki, Finland

STUDY SYNOPSIS

Introduction: Arthroscopic subacromial decompression (ASD) is the most commonly performed surgical intervention for shoulder pain, yet evidence on its efficacy is limited. The rationale for the surgery rests on the tenet that symptom relief is achieved through removal of a bony acromial spur and the resulting decompression of the tendon passage. Acknowledging the potential placebo effect of surgery, the primary objective of this superiority trial is to compare the efficacy of ASD versus diagnostic arthroscopy (DA) in patients with shoulder impingement syndrome (SIS), where DA differs only by the lack of subacromial decompression. As a non-surgical treatment option, a third group of supervised progressive exercise therapy (ET) will allow for pragmatic assessment of the relative benefits of surgical vs. non-operative treatment strategies.
Methods/Design: FIMPACT trial is an ongoing multicentre, three-group randomised controlled study with a primary objective of assessing the efficacy of the ASD vs. DA and a secondary objective of comparing ASD to exercise therapy (ET) in a pragmatic setting. We performed two-fold concealed allocation, first by randomising patients to surgical (ASD or DA) or conservative (ET) treatment in 2:1 ratio and then those allocated to surgery further to ASD or DA in 1:1 ratio. Our two primary outcomes are pain at rest and arm activity assessed with visual analog scale (VAS), while the secondary outcomes are functional assessment (Constant score and Simple shoulder test), quality of life (15D and SF-36), patient satisfaction, proportions of responders and non-responders, reoperations/treatment conversions, all at 2 years post-randomisation, as well as adverse effects and complications. We recruited a total of 210 patients from 3 tertiary referral centres. We will conduct the primary analysis on the intention-to-treat basis.

TRIAL REGISTRATION

ClinicalTrials.gov NCT00428870 (first registered January 29, 2007).

STUDY OBJECTIVES AND OUTCOMES

This statistical analysis plan (SAP) is accompanying the actual study protocol of the FIMPACT trial, a document that elaborates the methods used in detail. All outcomes were inquired from participants at baseline and follow-ups (6 and 24 months) and selected additional measures at 3 and 12 months (for details, see Table 1). The last patient reached the primary endpoint, the 24-month follow-up, in September 2015.

Table 1. Outcomes and follow-up time points

Assessment	Screening	Enrolment (Baseline)	Surgery	3 Months	6 Months	12 Months	24 Months	5 years	10 years
Screening form	X								
Informed consent		X							
Baseline characteristics form		X							
X-ray and MRI	X								X
Randomisation		X (1st)	X (2nd)						
Arthroscopic findings form			X						
Follow-up form*				X		X			
Clinical examination		X			X		X	X	X
Complications/adverse effects form**			(X)	(X)	(X)	(X)	(X)	(X)	(X)
VAS, at rest		X		X	X	X	X	X	X
VAS, at arm activity		X		X	X	X	X	X	X
Constant- Murley Score		X			X		X	X	X
Simple Shoulder Test (SST)		X			X		X	X	X
SF-36		X		X	X	X	X	X	X
15D		X		X	X	X	X	X	X
Return to work				X	X	X	X	X	X
Return to previous leisure activities				X	X	X	X	X	X
Responder analysis				X	X	X	X	X	X
Patients satisfaction to the treatment				X	X	X	X	X	X
Patients assessment of the treatment allocation				X					
Health resource utilisation				X	X	X	X	X	X

* Letter/telephone interview
** If required

DESCRIPTIVE OUTCOMES

At screening, the participants filled out a questionnaire to record gender, age, hand dominance, weight, height, level of education (socioeconomic status), workload (type of work), physical activity level, sports discipline, subjective health, symptoms (onset, frequency, and severity), use of pain medications, prior treatments, expectations to treatment, generic health state, and disease-specific scores. To exclude patients with concomitant shoulder pathology (particularly rotator cuff rupture), magnetic resonance imaging with contrast (MRA) was acquired for each participant.

OBJECTIVES AND PRIMARY OUTCOME

The primary objective of this trial is to compare the efficacy of arthroscopic subacromial decompression (ASD) versus diagnostic arthroscopy (DA) in patients with SIS. The trial is designed as a superiority trial, i.e. we expected in the power calculation that the ASD will result in greater pain relief at 24-month follow-up than DA (or ET). The 24-month follow-up was chosen as the primary endpoint, since this time point is a commonly held "minimal requirement" for any procedure in the field (orthopaedics) and most commonly used in the trials assessing the treatment of SIS.

The primary hypothesis: The primary hypothesis of our FIMPACT trial is that ASD is superior to DA in patients with SIS.

To enable pragmatic assessment of the relative benefits of surgical vs. non-operative treatment strategies on SIS, a non-surgical (third) treatment option of supervised progressive exercise therapy (ET) is also included (ASD vs. ET).

Additional hypothesis: The relative benefits of ASD and ET will be assessed without a priori hypothesis on the superiority of one or the other.

As the primary outcome measure, a visual analogue scale (0-100) was used to measure the patient's perceived pain intensity at rest and at arm activity during the 24 hours preceding the assessment. We considered 15 as the minimal clinically important difference (MCID) for SIS.[1]

SECONDARY OUTCOMES

Our secondary outcome measures are listed below. These outcomes will only be supportive, explanatory and/or hypothesis generating, which is why multiplicity is not considered to be a problem[2].

Constant-Murley score

Constant-Murley score (CS) is the most commonly used scoring system for evaluation of various disorders of the shoulder[3]. It consists of both objective (range of motion and strength) and subjective measurements (pain assessment, work load, and leisure time activities), which are summarised in a score between 0 and 100. A higher score indicates better shoulder function. The minimal clinically important improvement (MCII) of the Constant score is 17 for patients with SIS[4].

In addition, as night pain is considered one of the hallmark symptoms in patients with SIS and our two primary outcome measures (patient's perceived pain intensity at rest and at arm activity in the last 24 hours) do not specifically address this issue, a specific question from the Constant-Murley score (unaffected sleep: "Yes" or "No") will be analysed separately.

SST

The simple shoulder test (SST) was developed to assess any impairment of the patient's activities of daily living[5]. The SST consists of 12 questions with yes (1) or no (0) response options. The maximum SST score is 12 indicating normal shoulder function, minimum score of 0 points refers severely diminished shoulder function. The SST has good reliability and responsiveness in patients with rotator cuff symptoms[6]. The MCID for the SST in rotator cuff disease is 2 points[7].

15D

The 15D instrument is a generic health-related quality of life (HRQoL) instrument comprising 15 dimensions[8]. For each dimension, the respondent must choose one of the five levels that best describes his/her state of health at that the moment (the best level being 1 and the worst level being 5). A set of utility or preference weights is used in an addition aggregate formula

to generate a single index number, the utility or 15D score. The maximum 15D score is 1 (no problems on any dimension) and the minimum score is 0 (being dead). The responsiveness, reliability and validity of 15D have been thoroughly established, and this instrument has been used extensively in clinical and healthcare research[9 10].

SF-36

Short form or SF-36 is a generic HRQoL instrument to quantify the physical, functional, and psychological aspects of health-related quality of life. It consists of 36 questions in eight subscales that assess physical, functional, social, and psychological well-being[11]. Score ranges from 0 to 100, where a higher score is associated with better health. The physical and mental component summary scales (PCS and MCS, respectively) are then calculated as composites of the related subscales. SF-36 is one of most widely used measure of health-related quality of life[12].

Patient satisfaction and Responder analysis

We elicited patients' global assessment of satisfaction to the treatment with this question: "Are you satisfied with the treatment you have received?" We used a VAS scale ranging from 0 (completely disappointed) to 100 (completely satisfied).

Additionally, we elicited patient satisfaction to the treatment outcome with the following question at each follow-up time point (Table 1): "How satisfied are you with the outcome of your treatment?" on a 5-item scale. Participants who reported very satisfied or satisfied will be categorised as "Responders" and patients who responded very dissatisfied or dissatisfied as "Non-responders".

Return to previous leisure activities

Similarly, at each follow-up (Table 1), participants were asked to respond to the following question: "Have you been able to return to your previous leisure activities?" ("yes" or "no").

Patients' perception of operative treatment-group assignment

At the 3-month follow-up point, the patients in the two operative groups were asked to guess whether they had undergone ASD or DA.

Health resource utilisation and costs

For the cost-effectiveness analysis, at each follow-up visit the participants were asked to fill in a questionnaire inquiring about the use of healthcare resources. The questionnaire contains a list of items of healthcare resources available and the participants were asked to fill in the number of visits per item during the recall period of each follow-up time point. The resource use will be calculated based on the number of visits times unit cost per item and expressed as mean costs by items of resource use, and the mean direct total health care resource costs. All costs will be discounted to the 2016 price level.

Time to return to work

Information about return to work was recorded at each follow-up time point (Table 1).

Complications and adverse effects

Complications directly related to the interventions were registered. The participants were also encouraged to contact the participating hospitals if any adverse effects occurred and contacts to the health care system were monitored at every follow-up visit. Potential adverse effects (AE) were categorised to serious ad-verse effects (SAE) and minor adverse effects (MAE) if the participants sought treatment. Death, cardio-vascular or gastrointestinal effects, deep venous thrombosis, pulmonary embolism, systemic or local infection were categorised as SAEs and shoulder symptoms like pain, swelling and decreased range of motion were categorised as MAEs. The number and severity of complications and adverse effects will be assessed.

EXPLORATORY OUTCOMES

We have identified three potentially important effect modifying factors. We will perform subgroup analyses with the primary endpoint as the outcome and the direction of hypothesised effect described as below[14].

Duration of symptoms

We will compare the treatment effects stratified based on the duration of symptoms (those with < 6/12 months vs. those > 6/12 months). We hypothesise that subacromial decompression will work better in patients with duration of symptoms > 6 months than for patients with symptoms < 6 months.

Severity of symptoms

We will compare the treatment effects in patients with severe (VAS 70 or more), moderate (VAS 55 to 69), and mild (VAS less than 55) symptoms at baseline. We hypothesise that subacromial decompression will work better in patients with more severe (VAS 70 or more) than moderate (VAS 55 to 69) or mild (VAS less than 55) symptoms at baseline.

Acromial anatomy

We will compare the treatment effects in patients with flat (type I), curved (type II), or hooked (type III) acromion according to classification by Bigliani et al.[15] We hypothesise that subacromial decompression will work better in patients with hooked (type III) than curved (type II) or flat (type I) acromion at baseline.

STUDY DESIGN

Sample size

The sample size calculation was based on the two primary outcome measures, VAS at rest and at arm activity, at 24 months post randomisation. FIMPACT trial was powered to detect a minimal clinically important improvement (MCII) in a VAS pain score (improvement of at least 15; assumed standard deviation 25) between ASD and DA (or ET). To achieve a somewhat unconventional (stringent) 90% study power and using a two-sided Type I error rate (5%), our trial requires 68 patients per study group to show clinically meaningful advantage of ASD over DA (or ET). Acknowledging the stringent power threshold, only 3% surplus was reserved for potential loss to follow up/crossovers (3%), and accordingly, the recruitment target was set at 70 patients per treatment group.

Randomisation and blinding

To obtain three balanced study groups (of similar group size), we performed a two-fold, sequential randomisation. In Phase I, the participants were randomised into non-surgical or surgical treatment with allocation ratio 1:2. In the Phase II, those allocated to surgical treatment were further randomised to ASD or DA with 1:1 ratio. An independent statistician with no clinical involvement in the execution of the trial prepared separate randomisation lists for each study centre using a computer-generated algorithm. Randomisation was carried out using sequentially numbered sealed opaque envelopes. The envelopes were kept in a secure, agreed location at each centre. To ensure concealment, block randomisation was applied using blocks varying in size randomly, the block size known only by the statistician.

To initially enter a participant into the study (Phase I), an envelope containing the treatment assignment [non-surgical (ET) or surgery (ASD or DA), ratio 1:2] was opened during the baseline appointment. Participants randomised to ET started standardised physiotherapy within 2 weeks of the baseline appointment. Participants allocated to surgical treatment were scheduled for surgery aimed to be completed within 12 weeks of randomization.

At the day of surgery, an arthroscopic examination was first carried out to confirm the eligibility of the participant (to rule out full-thickness RC tear and other obvious intra-articular pathology). Research/staff nurse then completed the randomisation procedure (Phase II) by opening an envelope containing the surgical treatment allocation (ASD or DA, ratio 1:1). The allocation was revealed to the surgeon by showing the paper, but not expressed verbally.

The full follow-up process is shown in figure 1. In brief, the participants filled in the above noted (mailed) outcome questionnaires at 3, 6, 12 and 24 months post randomisation, in addition to which they were also assessed clinically at 6 and 24 months (and 5 and 10 years) post randomisation by a study physiotherapist unaware of treatment allocation, treatment given or possible unblinding. Outcome assessors were instructed not to inquire anything about prior treatment. Further, participants wore a t-shirt on all follow-up examinations. Data analysis will be done in a blinded manner by the study statistician (JR) not directly involved in the study.

STUDY POPULATION

Subject disposition

Study procedures, including recruitment strategies and inclusion and exclusion criteria, are presented in detail in the accompanying actual study protocol.

STATISTICAL ANALYSIS

Data will be analysed in a blinded manner. All p-values will be reported to 3 decimal places with those less than 0.001 reported as p < .001. The criterion for statistical significance will be set at alpha = 0.05.

Primary analysis

The primary analysis will be carried out according to the intention-to-treat (ITT) principle: participants are retained in the groups to which they were initially randomised. The primary comparison on the efficacy of ASD (ASD vs. DA) will be performed as a between-group comparison using a repeated measures mixed-effects model (RMMM). Study group and time of assessment (baseline, 3, 6, 12 and 24 months) will be included as fixed factors and patient as a random factor. The model will include interactions between study group and time of assessment. The baseline value will be included as a covariate. An unstructured covariance structure will be assumed. If the model cannot be fitted, compound symmetry will be assumed instead. The number of degrees of freedom will be assessed using Satterthwaite's method. The RMMM model will be used to quantify the treatment effect as the difference between the groups in pain scores (VAS) with the associated 95% confidence interval (CI) and p-value at 24 months post-primary randomisation. To safeguard against potential multiplicity bias[2], we will require a statistically significant treatment effect on both of our primary outcome variables, i.e., pain at rest and pain at activity (Table 2).

Table 2 Primary comparison ASD vs ET: Outcomes of the trial at 24 months follow-up.

	ASD	ET	Improvement from baseline		Between-Group Difference in Improvement from Baseline
**Primary outcomes**			ASD	ET	
VAS (rest)					
VAS (at arm activity)					
**Secondary outcomes**					
Constant-Murley Score					
SST					
SF-36					
15D					
Time to return to work					
Return to previous leisure activities					
Responder analysis					
Patients satisfaction to the treatment					
Patients assessment of the treatment allocation					
Complications and adverse effects					

Abbreviations: VAS, visual analogue scale; SST, Simple Shoulder Test; SF-36, Short form- 36

The same statistical model will also apply to the pragmatic comparison of the relative benefits of surgical vs. non-operative treatment strategies on SIS (ASD vs. ET) (Table 3).

Table 3 Secondary comparison ASD vs ET: Outcomes of the trial at 24 months follow-up.

Primary outcomes	ASD	ET	Improvement from baseline ASD	Improvement from baseline ET	Between-Group Difference in Improvement from Baseline
VAS (rest)					
VAS (at arm activity)					
**Secondary outcomes**					
Constant-Murley Score					
SST					
SF-36					
15D					
Time to return to work					
Return to previous leisure activities					
Responder analysis					
Patients satisfaction to the treatment					
Patients assessment of the treatment allocation					
Complications and adverse effects					

Abbreviations: VAS, visual analogue scale; SST, Simple Shoulder Test; SF-36, Short form- 36

Secondary analyses

We will also use the RMMM model to analyse secondary outcomes (Table 2 and 3) where applicable. The results will be reported as the differences between the groups with the associated 95% confidence interval (CI) and p-value at 24 months post-primary randomisation.

Furthermore, instead of focusing only on the statistical significance of the mean differences between treatment groups in the VAS (i.e., the mean improvement from baseline to 24 months), we will also carry out "a responder analysis". In principle, this analysis allows physicians to inform a patient of his or her chance of experiencing a clinically meaningful improvement from the treatment, both in absolute terms and in comparison, to a control group. The difference between responders and non-responders can be considered the net-benefit of the treatment. One proposed means to carry out a responder analysis relies on the assessment of the proportion of patients reaching the patient-acceptable symptom state (PASS) and the patient-disappointing symptoms state (PDSS). As no universal consensus exists on either the PASS or the PDSS in the context of SIS, we chose to anchor our responder analysis to the patient's assessment of satisfaction with the shoulder treatment outcome: Patients reporting very satisfied or satisfied will be categorised as "Responders" and those reporting very dissatisfied or dissatisfied as "Non-responders". Given the obvious coarseness of this approach, we plan to evaluate the appropriate criteria for PASS and PDSS in more detail in the future, exploring the potential contribution of, e.g., arm pain at rest and at activity, shoulder function, and night pain.

Categorical variables, the rates of unblinding, reoperation, treatment conversion, complications and adverse effects will be analysed using logistic regression analysis or Poisson regression dependent on whether subjects with complications or (multiple) complications (per subject) are analysed.

These secondary analyses will be supportive, explanatory and/or hypothesis generating, which is why multiplicity is not a problem[2].

Sensitivity analyses
The following two sensitivity analyses will be carried out: 1) per-protocol analysis, in which the above noted primary analyses will be carried out again with patients who received the interventions as allocated will be redone; 2) and potential effects due to the treatment providing centres.

As all the participants in the ASD group have received the critical therapeutic element (subacromial decompression), no treatment group conversion is possible in this group.

In the per-protocol comparison of the efficacy of ASD (ASD vs. DA), we define the DA per-protocol population as those participants who have not received ASD during the 24-month follow-up (who have not crossed over to ASD).

In the per-protocol comparison of the effectiveness of ASD (ASD vs. ET), we define the ET per-protocol population as those participants who have not received ASD during the 24-month follow-up (who have not crossed over to ASD).

INTERPRETATION OF RESULTS

To safeguard against potential risk of bias during interpretation, a method of "blinded data interpretation" will be used[17]. In brief, an independent statistician will provide the Steering/Writing committee of the FIMPACT trial with blinded results from the analyses with study groups labelled as group A, group B, and group C. This data will be presented to the Steering/Writing Committee, who will then contemplate on the interpretation of the results until a consensus is reached and agree in writing on all alternative interpretations of the findings. Once reaching a consensus, we will record the minutes of this meeting as a statement of interpretation document signed by all members of the Writing Committee. Only after reaching this common agreement will the data manager and independent statistician break the randomisation code.

There was also variation in the actual execution of the follow-up assessments, particularly in the earlier time-points (3- and 6-month follow-up visits).

IMPLEMENTATION OF ANALYSIS PLAN

This SAP will be used as a work description for the statistician performing the analyses. All analyses will be performed by the same statistician and none of the investigators involved in this trial will perform any of the statistical analyses.

The implementation of the SAP will be as follows:

1. A 'data collection form' will be outlined in a collaboration between the database manager (Leena Caravitis), statistician and principal investigators (Mika Paavola and Teppo Järvinen).

2. The database manager will code each treatment arm into 'treatment A', 'treatment B' and 'treatment C', thus leaving all others blinded to group assignment during the analyses.

3. Blinded data will be delivered to the statistician according to the 'data collection form'.

4. Primary, secondary and exploratory endpoint analyses will be made blinded to group assignment.

5. Results will be presented to the trial Writing and Steering committee, any uncertainties will be clarified and blinded interpretations of the primary endpoint results will be conducted prior to unblinding of data.

REFERENCES

1. Tashjian RZ, Deloach J, Porucznik CA, et al. Minimal clinically important differences (MCID) and patient acceptable symptomatic state (PASS) for visual analog scales (VAS) measuring pain in patients treated for rotator cuff disease. Journal of shoulder and elbow surgery / American Shoulder and Elbow Surgeons [et al] 2009;**18**(6):927-32.
2. Ranstam J. Multiple P-values and Bonferroni correction. Osteoarthritis Cartilage 2016;**24**(5):763-4.
3. Constant CR, Murley AH. A clinical method of functional assessment of the shoulder. Clinical orthopaedics and related research 1987(214):160-4.
4. Henseler JF, Kolk A, van der Zwaal P, et al. The minimal detectable change of the Constant score in impingement, full-thickness tears, and massive rotator cuff tears. Journal of shoulder and elbow surgery / American Shoulder and Elbow Surgeons [et al] 2015;**24**(3):376-81.
5. Matsen FA, 3rd, Ziegler DW, DeBartolo SE. Patient self-assessment of health status and function in glenohumeral degenerative joint disease. J Shoulder Elbow Surg 1995;**4**(5):345-51.
6. Godfrey J, Hamman R, Lowenstein S, et al. Reliability, validity, and responsiveness of the simple shoulder test: psychometric properties by age and injury type. J Shoulder Elbow Surg 2007;**16**(3):260-7.
7. Tashjian RZ, Deloach J, Green A, et al. Minimal clinically important differences in ASES and simple shoulder test scores after nonoperative treatment of rotator cuff disease. J Bone Joint Surg Am 2010;**92**(2):296-303.
8. Sintonen H. The 15D instrument of health-related quality of life: properties and applications. Ann Med 2001;**33**(5):328-36.
9. Moock J, Kohlmann T. Comparing preference-based quality-of-life measures: results from rehabilitation patients with musculoskeletal, cardiovascular, or psychosomatic disorders. Qual Life Res 2008;**17**(3):485-95.
10. Bowling A. *Measuring health. A review of quality of life measurement scales.* 3rd ed: Berkshire: Open University Press, 2004.
11. Ware JE, Jr., Sherbourne CD. The MOS 36-item short-form health survey (SF-36). I. Conceptual framework and item selection. Med Care 1992;**30**(6):473-83.
12. Garratt A, Schmidt L, Mackintosh A, et al. Quality of life measurement: bibliographic study of patient assessed health outcome measures. Bmj 2002;**324**(7351):1417.
13. Hamilton DF, Lane JV, Gaston P, et al. What determines patient satisfaction with surgery? A prospective cohort study of 4709 patients following total joint replacement. BMJ Open 2013;**3**(4).
14. Sun X, Ioannidis JP, Agoritsas T, et al. How to use a subgroup analysis: users' guide to the medical literature. Jama 2014;**311**(4):405-11.
15. Bigliani LU MD, April EW The morphology of the acromion and its relationship to rotator cuff tears. Orthop Trans 1986;**10**:228.
16. Little RJA, Rubin DB. *Statistical Analysis with Missing Data*. New York: John Wiley & Sons, Inc., 1987.
17. Jarvinen TL, Sihvonen R, Bhandari M, et al. Blinded interpretation of study results can feasibly and effectively diminish interpretation bias. J Clin Epidemiol 2014;**67**(7):769-72.

Minutes of the Blinded Data Interpretation Meeting

Interpretation of 1Blinded Data, Statement of Interpretation

National Institute for Health and Welfare, Center for Health and Social Economics, Helsinki, Finland

Date: December 7, 2016, at 8.30.

Present:

Jonas Ranstam, chair
Mika Paavola
Teppo Järvinen, secretary
Simo Taimela
Antti Malmivaara
Kari Kanto
Pirjo Toivonen
Leena Caravitis
Jari Inkinen

Background assumptions regarding our primary comparison (ASD vs. DA)

1) This superiority RCT is designed to address the true *efficacy* of arthroscopic subacromial decompression (ASD), i.e., can ASD theoretically work? Accordingly, we have chosen patients that – based on the existing literature – represent optimal responders to this index surgical procedure.
2) Conceding that the act of surgery *per se* produces a profound placebo response, a 'true' treatment effect is impossible to disentangle from the nonspecific (placebo or meaning) effects – such as the patients' or researchers' expectations of benefit – without a placebo comparison group.
3) The only difference between ASD and DA treatment groups is that the subacromial decompression, the critical therapeutic (surgical) element, has been carried out for patients in the ASD group.
    a. The critical therapeutic (surgical) element is the component of the surgical procedure that is believed to provide the therapeutic effect (here, subacromial decompression), being distinct from aspects of the procedures that are diagnostic or required to access the disease being treated (here, shoulder arthroscopy).
    b. Apart from the critical therapeutic element, the treatment of the ASD and DA groups is identical, i.e., all "placebo or meaning effect" related to the entire treatment and care is identical.
4) To be deemed effective, ASD should provide a statistically significant benefit over DA in both of the two primary outcomes, pain at rest and activity assessed with a visual analog scale (VAS), as determined by the mean VAS difference between the groups. This is to safeguard against potential multiplicity bias[2].

If ASD is found effective (see above), it should also provide a clinically relevant benefit over DA according to following rationale:

1) There is a proven benefit as follows: Mean VAS-difference between ASD and DA shall exceed the threshold for the minimal clinically important difference (MCID) in VAS. We will consider 15 as the threshold for the minimal clinically important difference (MCID).

AND,

2) There is NO proven harm. If there is a proven benefit of ASD but significantly higher proportion of patients show adverse effects, the amount of benefits will be discussed in relation to the frequency and seriousness of the adverse effects.

Statistical commitments:

a) I-T-T is the primary data analysis, but per-protocol analysis will also be carried out.
b) The pre-specified time point of primary interest is 24 months after randomisation.
c) In addition to the two primary outcome parameters, we will also take into account the number of treatment conversions and re-operations, the incidence and seriousness of adverse effects between the ASD and DA groups, and the responder analysis.

Based on these theoretical commitments, our interpretation of the findings will be as follows:

a) If ASD is found superior to DA, the critical therapeutic element of the ASD procedure (subacromial decompression) has a clinically relevant effect on patients with symptoms consistent with SIS.
b) If ASD is not found superior to DA, the critical therapeutic element of the ASD procedure (subacromial decompression) does not have a clinically relevant effect on patients with symptoms consistent with SIS. Considering our efficacy design (study participants are 'optimal responders to ASD' and the surgeons are highly experienced), such finding would imply that ASD does not work at all.

Minutes of the "Blinded review of the data"

The Steering and Writing Committees of the FIMPACT-trial (undersigned, below) developed and recorded three interpretations of the results on the basis of a blinded review of the primary outcome data (treatment A compared with treatment B, treatment A compared with treatment C, and treatment B compared with treatment C).

Jonas Ranstam had the groups coded as follows:

A = 1

B = 2

C = 3

While reviewing the blinded results of the three comparisons, the undersigned noted that in the two primary outcomes, the treatment group mean values were virtually identical at baseline in all three groups (below). Also, it was noted that the VAS at rest values were slightly above 40 points, while the VAS during activity were slightly above 70 points, again virtually identical.

Table 1: Baseline characteristics

Pain measurements/Shoulder scores

	Group A	Group B	Group C
Pain at rest (0-100 VAS scale), mean (SD)	41.7 (27.5)	41.3 (25.8)	41.6 (25.5)
Pain during activity (0-100 VAS scale), mean (SD)	72.4 (20.8)	71.2 (23.6)	72.3 (21.7)

Most importantly, the undersigned noted that the only statistically significant between-group difference in the primary analysis was in a comparison between treatment A and treatment B.

1. Accordingly, our interpretations between the comparisons A vs. C and B vs. C - irrespective of whether they represent our primary comparison (ASD vs. DA) or secondary comparison (ASD vs. ET) – are as follows:

a) Treatment A vs. Treatment C

Our data suggest that both treatments result in a marked improvement in pain over time (both at rest and during activity), but there is no empirical evidence for a differential treatment effect.

b) Treatment B vs. Treatment C

Our data suggest that both treatments result in a marked improvement in pain over time (both at rest and during activity), but there is no empirical evidence for a differential treatment effect.

2. The only *statistically* significant difference was found between treatment A and treatment B in both primary outcome measures in the primary analyses (MMRM ANOVA, over the course of 24 month follow-up) (Document: Results from an analysis of the FIMPACT trial by Jonas Ranstam).

According to our pre-specified (submitted, under revision in the BMJ Open: referred as "Protocol" from now on) scheme for blinded data interpretation, a statistically significant between-group difference requires an analysis on the clinical relevance. For this analysis, we have set a pre-determined threshold based on the minimal clinically important difference, MCID: The between-group difference between treatment A and treatment B has to exceed 15 points in the two primary outcomes.

Our results show a between-group difference of -7.5 (95% CI: -14 to -1, p=0.0231) and -12.1 (95% CI: -21 to -3, p=0.0075) in VAS at rest and VAS during activity, respectively. Thus, this analysis shows that the confidence interval of the latter outcome (VAS during activity) includes a clinically relevant difference, although the mean difference does not exceed the threshold for the MCID.

The participants contemplated on the possible clinical relevance of the observed between-group difference and agreed on the interpretation of the observed difference between Treatment A and Treatment B stands as follows:

If Groups A and B are Diagnostic arthroscopy (DA) and Exercise therapy (ET), irrespective of the order, the analysis will be ignored as clinically-irrelevant.

If this comparison (treatment A vs. treatment B) proves to be our primary (ASD vs. DA) or secondary (ASD vs. ET) comparison, our interpretation of the findings is as follows:

If Groups A and B are Arthroscopic subacromial decompression (ASD) and Diagnostic arthroscopy (DA), our interpretation concerns the primary comparison and is as follows:

Primary comparison: ASD vs. DA

a) If Group A = Arthroscopic subacromial decompression, ASD and Group B = Diagnostic arthroscopy, DA

Our results suggest that ASD results in a slightly inferior benefit in shoulder pain over DA, and subsequently, diminishes the tolerability to activity. Given that the only difference between the two groups is the critical therapeutic element, this analysis proves that ASD is clinically futile.

b) If Group A = Diagnostic arthroscopy, DA and Group B = Arthroscopic subacromial decompression, ASD
Our results suggest that ASD results in a slightly superior benefit in shoulder pain over DA, and subsequently, increases the tolerability to activity. Given that the only difference between the two groups is the critical therapeutic element, this analysis proves that ASD has some benefit, but the clinical relevance of the finding requires further consideration (see below).

If Groups A and B are Arthroscopic subacromial decompression (ASD) and Exercise therapy (ET), our interpretation concerns the secondary comparison and is as follows:

Secondary comparison: ASD vs. ET

a) If Group A = Arthroscopic subacromial decompression, ASD and Group B = Exercise therapy, ET
Our results suggest that ASD results in a slightly inferior benefit in shoulder pain over ET, and subsequently, diminishes the tolerability to activity. This analysis will be interpreted as evidence that ET should be preferred over ASD.

b) If Group A = Exercise therapy, ET and Group B = Arthroscopic subacromial decompression, ASD
Our results suggest that ASD results in a slightly superior benefit in shoulder pain over ET, and subsequently, increases the tolerability to activity. This analysis will be interpreted as evidence that ASD could have a role in the treatment of patients with SIS, however the clinical relevance of the finding requires further consideration, including the pre-specified sensitivity analysis (see Protocol/Statistical Analysis Plan, SAP).

After reaching a consensus on the interpretation of the primary and secondary comparisons, we proceeded into secondary analysis of the data.

Secondary analysis of the data (hypothesis-generating)

A. Analyses basing on our two primary outcomes (VAS at rest and during activity)

The participants of the meeting agreed that the VAS at rest values at baseline were relatively low in both groups, while the corresponding VAS during activity were high, further supporting the inference that VAS during activity represents a more clinically relevant outcome and justifies further analysis.

According to our pre-specified blinded data interpretation scheme, we proceeded into analysis of harms. Our premise was that if the proven benefit of treatment B comes at the cost of significantly higher proportion of participants showing adverse effects and/or ending up with a disappointing outcome, the benefit is weighted in relation to the frequency and seriousness of these negative effects.

B. Analyses basing on our secondary outcomes

The analysis of harms was two-fold: we assessed a) complications and adverse effects related to both treatment group A and B, and b) Responder analysis (satisfaction with outcome of treatment), as specified in our Protocol.

Both analyses were carried out blinded to the treatment groups.

a) We noted that the incidence of complications and adverse effects were highly comparable between the two treatment groups (A and B), not materially affecting our interpretation (preference of one treatment over another).

b) Responder analysis showed no statistically significant difference between the treatment A and B.

Other secondary outcomes:

The only statistically significant between-group differences in the secondary outcomes were observed in the Constant-Murray score (Treatment B > A) and SF-36 Mental Health –subscale (Treatment A > B and Treatment C > B).

A consensus was reached that these findings have no influence on our primary interpretations.

Finally, the randomization code was broken (at 13.52), revealing the following group assignments.

A = 1 Exercise therapy
B = 2 Arthroscopic subacromial decompression
C = 3 Diagnostic arthroscopy

Final interpretation, after knowledge of the group assignments:

Primary comparison: ASD (Group 2) vs. DA (Group 3)

Our results suggest that the critical therapeutic element of the ASD procedure (subacromial decompression) does not have a clinically relevant effect on patients with symptoms consistent with SIS.

Secondary comparison: ASD (Group 2) vs. ET (Group 1)

Our results suggest that ASD results in a slightly superior benefit in shoulder pain over ET, and subsequently, increases the tolerability to activity. This analysis will be interpreted as evidence that ASD could have a role in the treatment of patients with SIS. However, as noted in our Blinded data interpretation scheme, this finding is subject to possible biases (see below). Final interpretation can only be made after carrying out the pre-specified sensitivity analyses (see Protocol/Statistical Analysis Plan, SAP).

Background assumptions regarding our secondary (independent) comparison (ASD vs. ET)

1) This pragmatic comparison is designed to address whether arthroscopic subacromial decompression (ASD) followed by postoperative rehabilitation is superior to supervised progressive exercise therapy (ET). We recognize that in this pragmatic comparison (ASD vs. ET) the supervised progressive exercise therapy regimen carried out in the ET group is different from the postoperative rehabilitation carried out by patients in the ASD group. In addition, the timing of the start of the actual treatment between the ET and ASD groups was somewhat discrepant due to the time required to arrange the surgery. The ASD patients are also subject to

some degree of postoperative immobilization, sick-leave, and modification of pain medication and activities, unlike the patients in the ET group, all of which may also have an effect on the treatment outcome. However, these concord with the current best practice recommendations and the two-year follow-up chosen as our primary time point should dilute the effects of somewhat discrepant timing of the interventions.

2) To be deemed effective, either ASD or ET should provide a statistically significant benefit over ET or ASD, respectively, in both of our two primary outcomes, pain at rest and activity assessed with a visual analog scale (VAS), as determined by the mean VAS difference between the two treatment groups. This is to safeguard against potential multiplicity bias[2].
3) The following concern (apparent confounding) needs to be taken into account in the interpretation. Despite the thorough *preoperative* screening, 10% (14/136) allocated to the two surgical groups had to be excluded because of pathology found after the 1st random allocation. Although this does not have any effect on our primary comparison (ASD vs. DA), the ET and ASD groups are not fully comparable. This discrepancy will possibly skew our results by favouring the ASD group.

Acknowledging all this, if ASD (or ET) is found effective (statistically significant difference in both primary outcomes), it should also provide a clinically relevant benefit over ET (or ASD) according to following rationale:

1) There is a proven benefit as follows: Mean VAS-difference between ASD and ET shall exceed the threshold for the minimal clinically important difference (MCID) in VAS. We will consider 15 as the threshold for the minimal clinically important difference (MCID).

AND,

2) There is NO proven harm. If there is a proven benefit of ASD (or ET) but significantly higher proportion of patients show adverse effects, the amount of benefits will be discussed in relation to the frequency and seriousness of the adverse effects.

Statistical commitments:

a) I-T-T is the primary data analysis, but per-protocol analysis will also be carried out.

b) The pre-specified time point of primary interest is 24 months after randomisation.
c) In addition to the two primary outcome parameters, we will also take into account the number of treatment conversions and re-operations, the incidence and seriousness of adverse effects between the ASD and ET groups, and the responder analysis.
d) Given the Background assumption 3.a) (above, the discrepant number of patients with a shoulder pathology other than SIS), we will carry out a worst-case analysis by creating a subgroup of the ET group by removing seven (an equal number of patients excluded from both surgical treatment arms due to pathology found after 1st randomization) worst-cases/highest VAS-pain scores at the primary analysis time-point (24 months). The number of removed cases is based on the assumption that the prevalence of shoulder pathology is identical in the randomized population, while the decision to remove the individual with the highest VAS-pain scores at the end of the study basis on the assumption that shoulder pathology is an effect-modifying factor, predicting poor outcome.

Based on these theoretical commitments, our interpretation of the findings will be as follows:

a) If ASD is found superior to ET in both the complete case and the sensitivity (subgroup) analyses, ASD is a more effective treatment option than ET for patients with SIS.
b) If ET is found superior to ASD in both the complete case and the sensitivity (subgroup) analyses, our results suggests that ET is a more effective treatment option than ASD for patients with subacromial pain syndrome.
c) If there are no statistically significant differences between ASD and ET, ASD and ET are equally effective.

Jonas Ranstam, chair   Mika Paavola   Teppo Järvinen, secretary   Simo Taimela

Antti Malmivaara   Kari Kanto   Leena Caravitis   Pirjo Toivonen

Jari Inkinen
