# Supplementary material for: Subacromial decompression versus diagnostic arthroscopy for shoulder impingement: randomised, placebo surgery controlled clinical trial
Source: BMJ. 2018 Jul 19;362:k2860. doi: 10.1136/bmj.k2860 (PMC6052435; doi:10.1136/bmj.k2860)
Supplement: Supplementary file 2 — Appendix 2 [file paam040473.ww2.pdf]

# Supplementary appendix 2

## Table of Contents

Table S1 Inclusion and Exclusion Criteria 2
Table S2 Subgroup analysis on resection of the subacromial bursa within the DA group 3
Table S3 Trajectories of the primary outcomes at 3, 6 and 12 months (ASD vs. DA) 4
Table S4 Trajectories of the secondary outcomes at 3, 6 and 12 months (ASD vs. DA) 5
Table S5 Sensitivity analyses of the primary and secondary outcomes concerning the primary comparison (ASD vs. DA) 6
Table S6 Subgroup analyses concerning the primary comparison (ASD vs. DA) 7
Table S7 Unblindings, treatment conversions and reoperations 8
Table S8 Trajectories of the primary outcomes at 3, 6 and 12 months (ASD vs. ET) 9
Table S9 Trajectories of the secondary outcomes at 3, 6 and 12 months (ASD vs. ET) 10
Table S10 Sensitivity analyses of the primary and secondary outcomes concerning the secondary comparison (ASD vs. ET) 11
Table S11 Proportions of patients in the ASD and ET groups exceeding the threshold for minimal clinically important improvement (MCII) and reaching the patient-acceptable symptom state (PASS) 12
Table S12 Frequency of missing data at 3, 6, 12 and 24 months 13

This supplementary material has been provided by the authors to give readers additional information about their work.

**Table S1 Inclusion and exclusion criteria.**

| | |
|---|---|
| **Inclusion criteria** | |
| 1. | Adult men or women ages 35 to 65 years |
| 2. | Subacromial pain for greater than 3 months with no relief from non-operative means (physiotherapy, non-steroidal anti-inflammatory medication, corticosteroid injections, and rest) |
| 3. | Pain provoked by abduction and positive painful arc –sign |
| 4. | Positive impingement test (temporary relief of pain by subacromial injection of lidocaine) |
| 5. | Pain in at least 2 out of 3 of isometric tests (abduction 0° and 30° or external rotation) |
| 6. | Provision of informed consent from the participant |
| 7. | Ability to speak, understand and read in the language of the clinical site |
| **Exclusion criteria** | |
| 1. | Full thickness tear of the rotator cuff tendons diagnosed on clinical examination (marked weakness in any of the examined muscles) or magnetic resonance imaging with intra-articular contrast (MRA) |
| 2. | Osteoarthritis of the glenohumeral and/or acromioclavicular joint diagnosed on clinical examination and on x-rays |
| 3. | Substantial calcific deposits in the rotator cuff tendons found in the preoperative imaging |
| 4. | Previous surgical procedure on the affected shoulder |
| 5. | Evidence of shoulder instability (positive apprehension/positive sulcus sign) |
| 6. | Symptomatic cervical spine pathology |
| 7. | History of alcoholism, drug abuse, psychological or psychiatric problems that are likely to invalidate informed consent |
| 8. | Patient declined to participate |

**Table S2 Subgroup analysis on resection of the subacromial bursa within the DA group.**

During the arthroscopic examination, the rotator cuff (RC) integrity was evaluated from the subacromial space without performing routine bursectomy. However, if the integrity of the RC could not be verified otherwise, bursal tissue was bluntly stretched with troachar or resected on the tendon side to allow visualization. A resection was carried out in 18/63 of the DA participants: the resection was minimal in 15/63 and extensive in 3/63 of the participants. Given that one previous trial suggested a therapeutic effect for bursectomy (Henkus et al. 2009), we carried out a subgroup analysis on this potential effect within the DA group. The upper panel provides the *non-adjusted* mean (95% confidence intervals) values of the two primary outcomes in the different DA subgroups stratified based on the amount of resection carried out.

The lower panel provides the actual results of the subgroup analysis, the estimated effect expressed in VAS points. The no resection group was used as a reference and the estimated effect values indicate the difference between any given subgroup (comparator) and the reference. **A positive estimated effect means that the participants in the comparator (i.e., resection) subgroup reported a higher pain level at the 24-month time point.** The analyses were carried out using a mixed model repeated measures (MMRM) ANOVA *with adjustment for baseline imbalance*.

| | n* | Pain VAS at rest (Mean, 95% CI) | Pain VAS on arm activity (Mean, 95% CI) |
|---|---|---|---|
| No resection | 45 (42) | 7.3 (1.8 to 12.9) | 22.2 (13.9 to 30.5) |
| Minimal resection | 15 (14) | 7.9 (-1.3 to 17.0) | 22.8 (8.1 to 37.5) |
| Extensive resection | 3 (3) | 48.0 (-51.5 to 147.5) | 62.3 (-59.3 to 183.9) |
| Any resection (minimal + extensive) | 18 (17) | 14.9 (1.8 to 28.1) | 29.8 (13.0 to 46.6) |
| | **Estimated effect** | **95% Confidence interval** | **P value** |
| **Pain VAS at rest** | | | |
| Any resection vs. No resection | 3.6 | -1.7 to 8.9 | 0.16 |
| Minimal resection vs. No resection | 3.1 | -7.3 to 13.6 | 0.18 |
| Extensive resection vs. No resection | 3.5 | -1.4 to 8.5 | 0.56 |
| **Pain VAS on arm activity** | | | |
| Any resection vs. No resection | 6.2 | -2.8 to 15.1 | 0.17 |
| Minimal resection vs. No resection | 7.8 | -1.8 to 17.5 | 0.11 |
| Extensive resection vs. No resection | -1.8 | -20.9 to 17.3 | 0.85 |

* The n values in the upper panel refer to the number of participants in the different DA subgroups stratified based on the amount of resection carried out and the numbers within the parentheses represent the number of actual observations at the 24-month follow up.

**Table S3 Trajectories of the primary outcomes at 3, 6 and 12 months (ASD vs. DA).**

The table shows the mean values and between-group differences in VAS pain scores at the 3, 6 and 12-month follow-ups with 95% confidence intervals. The trajectories are estimated using a mixed-effects model repeated measures (MMRM) ANOVA with adjustment for baseline imbalance. N denotes the number of subjects.

| Outcomes | Arthroscopic subacromial decompression (ASD) | n | Diagnostic arthroscopy (DA) | n | Between-group difference ASD vs. DA |
|---|---|---|---|---|---|
| **3 months** | | | | | |
| Pain VAS at rest | 21.6 (16.9 to 26.3) | 54 | 19.8 (15.2 to 24.4) | 55 | 1.8 (-4.8 to 8.4) |
| Pain VAS on arm activity | 42.1 (35.3 to 48.8) | 52 | 37.4 (30.9 to 44.0) | 55 | 4.6 (-4.8 to 14.0) |
| **6 months** | | | | | |
| Pain VAS at rest | 15.9 (11.4 to 20.4) | 59 | 14.0 (9.6 to 18.4) | 61 | 1.9 (-4.3 to 8.2) |
| Pain VAS on arm activity | 37.9 (31.5 to 44.3) | 59 | 37.7 (31.5 to 44.0) | 61 | 0.1 (-8.8 to 9.1) |
| **12 months** | | | | | |
| Pain VAS at rest | 11.7 (7.1 to 16.3) | 55 | 13.3 (8.7 to 17.8) | 56 | -1.6 (-8.1 to 4.9) |
| Pain VAS on arm activity | 23.3 (16.7 to 29.9) | 55 | 28.1 (21.6 to 34.5) | 56 | -4.8 (-14.0 to 4.5) |

The between-group differences may not exactly equal the differences in change in the scores between the ASD and DA groups because of the adjustment for baseline imbalance in the MMRM analyses. A negative between-group difference means that the participants in the ASD group reported a lower pain level at the 24-month time point.

The minor discrepancies in the results of the ASD group in this Table S3 and in the Table S8 are due to the adjustment for baseline imbalance when analysing change from baseline. The adjustment differs based on which groups are included in the model.

**Table S4 Trajectories of the secondary outcomes at 3, 6 and 12 months (ASD vs. DA).**

The table shows the mean values and between-group differences in the secondary outcomes at the 3, 6 and 12-month follow-ups with 95% confidence intervals. The trajectories are estimated using a mixed-effects model repeated measures (MMRM) ANOVA with adjustment for baseline imbalance. N denotes the number of subjects.

| Outcomes | Arthroscopic subacromial decompression (ASD) | n | Diagnostic arthroscopy (DA) | n | Between-group difference ASD vs. DA |
|---|---|---|---|---|---|
| **3 months** | | | | | |
| Constant-Murley score | N/A | | N/A | | N/A |
| SST | N/A | | N/A | | N/A |
| Satisfaction to the treatment (0-100 VAS) | 76.9 (71.7 to 82.2) | 57 | 81.4 (76.3 to 86.6) | 57 | -4.5 (-11.9 to 2.9) |
| 15-D | 0.91 (0.90 to 0.92) | 55 | 0.92 (0.91 to 0.93) | 54 | -0.1 (-0 to 0) |
| **6 months** | | | | | |
| Constant-Murley score | 58.1 (53.9 to 62.4) | 59 | 64.3 (60.1 to 68.5) | 61 | -6.2 (-12.1 to 0.2) |
| SST | 7.9 (7.3 to 8.5) | 59 | 8.5 (7.9 to 8.0) | 61 | -0.5 (-1.4 to 0.3) |
| Satisfaction to the treatment (0-100 VAS) | 74.7 (69.5 to 80.0) | 56 | 79.6 (74.4 to 85.7) | 58 | -4.8 (-12.2 to 2.5) |
| 15-D | 0.92 (0.90 to 0.93) | 55 | 0.92 (0.91 to 0.94) | 55 | 0 (-0 to 0) |
| **12 months** | | | | | |
| Constant-Murley score | N/A | | N/A | | N/A |
| SST | N/A | | N/A | | N/A |
| Satisfaction to the treatment (0-100 VAS) | 82.7 (77.4 to 88.0) | 55 | 85.3 (80.0 to 90.6) | 54 | -2.6 (-10.1 to 4.9) |
| 15-D | 0.92 (0.91 to 0.94) | 54 | 0.93 (0.92 to 0.94) | 53 | 0 (-0 to 0) |

The between-group differences may not exactly equal the differences in change in the scores between the ASD and DA groups because of the adjustment for baseline imbalance in the MMRM analyses. N/A = not applicable. For all variables, a higher score indicates a better treatment outcome.

**Table S5 Sensitivity analyses of the primary and secondary outcomes concerning the primary comparison (ASD vs. DA).**

The table shows the between-group differences at the 24-month follow up. The estimated effect indicates the mean difference, ASD minus DA. The analyses were carried out using a mixed model repeated measures (MMRM) ANOVA with adjustment for baseline imbalance.

| Variable | ITT Estimated effect (95% CI)* | P value | Per protocol Estimated effect (95% CI)† | P value | As treated Estimated effect (95% CI)‡ | P value |
|---|---|---|---|---|---|---|
| Pain VAS at rest | -4.58 (-11.27 to 2.11) | 0.18 | -4.42 (-11.05 to 2.21) | 0.19 | -4.15 (-10.61 to 2.31) | 0.21 |
| Pain VAS on activity | -8.96 (-18.08 to 0.16) | 0.054 | -7.87 (-17.19 to 1.45) | 0.10 | -4.46 (-13.31 to 4.39) | 0.32 |
| Constant-Murley score | 4.25 (-1.99 to 10.49) | 0.18 | 2.36 (-3.88 to 8.60) | 0.46 | -1.34 (-7.34 to 4.66) | 0.66 |
| SST | 0.46 (-0.38 to 1.30) | 0.29 | 0.16 (-0.70 to 1.02) | 0.72 | -0.15 (-0.97 to 0.67) | 0.72 |
| Satisfaction to the treatment (VAS 0-100) | 0.88 ( -6.58 to 8.34) | 0.82 | 0.15 (-7.17 to 7.47) | 0.97 | -1.33 (-8.47 to 5.81) | 0.72 |
| 15-D | 0.00 (0.02 to -0.02) | 1.00 | 0.00 (0.02 to -0.02) | 1.00 | 0.00 (0.02 to -0.02) | 1.00 |

* ITT, the intention to treat population (ASD: n = 59, DA: n = 63).

† The per protocol population is the subset of the intention-to-treat population who received the treatment they were randomised to and who did not receive any other treatment, i.e. the patients with a treatment conversion have been excluded (ASD: n = 59, DA: n = 55).

‡ The as treated population is defined according to the treatment the participants received, i.e. the 8 participants who originally received DA and the 14 participants who originally received ET, but due to persistent symptoms requested unblinding and subsequently received ASD, have been included in the ASD population (ASD: n = 81, DA: n = 55).

**Table S6 Subgroup analyses concerning the primary comparison (ASD vs. DA).**

The table shows the between-group differences at the 24-month follow up by subgroups. The estimated effect indicates the mean difference, ASD minus DA. The analyses were carried out using a mixed model repeated measures (MMRM) ANOVA with adjustment for baseline imbalance by including the subgroup factor as a covariate, one at a time, in the model. The interaction test refers to the interaction *Subgroup factor* Treatment Group*. A negative effect estimate indicates lower pain in the ASD group.

| | Pain VAS at rest | | | | Pain VAS on activity | | | |
|---|---|---|---|---|---|---|---|---|
| **Subgroup factor** | Estimated effect (VAS) | 95% Confidence Interval | P value main effect | P value interaction test | Estimated effect (VAS) | 95% Confidence Interval | P value main effect | P value interaction test |
| **Duration of symptoms** | | | | | | | | |
| Symptoms <12mo. (n=55) | -6.70 | -13.96 to 0.56 | 0.071 | 0.245 | -12.15 | -23.16 to -1.14 | 0.031 | 0.324 |
| Symptoms ≥12mo. (n=63) | -2.72 | -9.76 to 4.32 | 0.449 | | -6.16 | -16.74 to 4.13 | 0.253 | |
| **Severity of symptoms** | | | | | | | | |
| VAS < 70 (n=39) | -0.50 | -8.40 to 7.40 | 0.901 | 0.097 | -7.06 | -19.56 to 5.44 | 0.268 | 0.679 |
| VAS ≥ 70 (n=79) | -6.49 | -13.22 to 0.23 | 0.058 | | -9.74 | -19.67 to 0.19 | 0.055 | |
| VAS <55 (n=22) | 3.25 | -6.24 to 12.73 | 0.502 | 0.093 | -5.15 | -20.80 to 10.50 | 0.519 | 0.844 |
| VAS 55 to 69 (n=17) | -3.10 | -14.09 to 7.89 | 0.581 | | -10.00 | -28.59 to 8.59 | 0.292 | |
| VAS ≥70 (n=79) | -6.50 | -13.22 to 0.21 | 0.058 | | -9.75 | -19.72 to 0.22 | 0.055 | |
| **Acromial anatomy (Bigliani classification)** | | | | | | | | |
| Type 1 and 2 (Flat and Curved) (n=107) | -5.16 | -11.57 to 1.25 | 0.115 | 0.52 | -8.84 | -18.07 to 0.39 | 0.060 | 0.775 |
| Type 3 (Hooked) (n=11) | -1.33 | -13.7 to 11.04 | 0.833 | | -11.82 | -32.51 to 8.87 | 0.263 | |
| Type 1 (Flat) (n=35) | 0.47 | -8.16 to 9.10 | 0.915 | 0.087 | -10.85 | -24.77 to 3.07 | 0.127 | 0.928 |
| Type 2 (Curved) (n=72) | -8.08 | -14.95 to -1.21 | 0.021 | | -8.63 | -18.94 to 1.68 | 0.101 | |
| Type 3 (Hooked) (n=11) | -1.31 | -13.55 to 10.93 | 0.834 | | -11.82 | -32.64 to 9.00 | 0.266 | |

**Table S7 Unblindings, treatment conversions, and reoperations.**

| Study group | Unblinding or decision on treatment conversion. Time from randomization (Months) | Treatment converted to* | Reoperations | Time from randomization to reoperation (months) |
|---|---|---|---|---|
| ET | 2 | ASD | | |
| ET | 5 | ASD | MUA and ADCR | 10 |
| ET | 6 | ASD and MUA | | |
| ET | 7 | ASD | | |
| ET | 7 | ASD | | |
| ET | 7 | ASD | ASD and LHBT | 23 |
| ET | 7 | ASD | | |
| ET | 7 | ASD | | |
| ET | 8 | ASD | | |
| ET | 8 | ASD | | |
| ET | 9 | ASD | ASD and ADCR | 57 |
| ET | 11 | ASD | | |
| ET | 12 | ASD | | |
| ET | 15 | ADCR | | |
| ET | 21 | ACR and ASD | | |
| ASD | 3 | | | |
| ASD | 8 | | | |
| ASD | 8 | MUA | | |
| ASD | 13 | | | |
| ASD | 14 | | | |
| ASD | 18 | ADCR | ASD and ADCR | 51 |
| DA | 1 | ASD | | |
| DA | 5 | ASD | | |
| DA | 6 | | | |
| DA | 9 | ASD | | |
| DA | 9 | ASD | | |
| DA | 9 | ASD | | |
| DA | 11 | ASD | | |
| DA | 12 | ASD | | |
| DA | 19 | ASD and SSC repair | | |

Abbreviations: ASD=Arthroscopic Subacromial Decompression; ACR=Arthroscopic Capsular Release; MUA=Manipulation Under Anesthesia; ADCR=Arthroscopic Distal Clavicle Resection; LHBT=Long Head of the Biceps Tendon; SSC repair=Subscapularis tendon repair.

* Treatment conversions (surgeries) were carried out within 4 months of unblinding (mean: 6 weeks, range 1-16 weeks).

**Table S8 Trajectories of the primary outcomes at 3, 6 and 12 months (ASD vs. ET).**

The table shows the mean values and between-group differences in VAS pain scores at the 3, 6 and 12-month follow-ups with 95% confidence intervals. The trajectories are estimated using a mixed-effects model repeated measures (MMRM) ANOVA with adjustment for baseline imbalance. N denotes the number of subjects.

| Outcomes | Arthroscopic subacromial decompression (ASD) | n | Exercise Therapy (ET) | n | Between-group difference, ASD vs. ET |
|---|---|---|---|---|---|
| **3 months** | | | | | |
| Pain VAS at rest | 21.7 (16.7 to 26.6) | 54 | 23.8 (19.2 to 28.4) | 62 | -2.2 (-8.9 to 4.6) |
| Pain VAS on arm activity | 42.3 (35.5 to 49.1) | 52 | 44.5 (38.3 to 50.8) | 62 | -2.2 (-11.5 to 7.0) |
| **6 months** | | | | | |
| Pain VAS at rest | 15.9 (11.2 to 20.7) | 59 | 16.9 (12.4 to 21.3) | 67 | -0.9 (-7.4 to 5.6) |
| Pain VAS on arm activity | 38.1 (31.7 to 44.6) | 59 | 44.6 (38.5 to 50.1) | 67 | -6.4 (-15.3 to 2.4) |
| **12 months** | | | | | |
| Pain VAS at rest | 11.7 (6.8 to 16.6) | 55 | 17.1 (12.6 to 21.7) | 65 | -5.5 (-12.1 to 1.2) |
| Pain VAS on arm activity | 23.5 (16.9 to 30.2) | 55 | 33.1 (26.9 to 39.3) | 64 | -9.6 (-18.6 to -0.5) |

The between-group differences may not exactly equal the differences in change in the scores between the ASD and DA groups because of the adjustment for baseline imbalance in the MMRM analyses. A negative between-group difference means that the participants in the ASD group reported a lower pain level at the 24-month time point.

The minor discrepancies in the results of the ASD group in this Table S8 and in the Table S3 are due to the adjustment for baseline imbalance when analysing change from baseline. The adjustment differs based on which groups are included in the model.

**Table S9 Trajectories of the secondary outcomes at 3, 6 and 12 months (ASD vs. ET).**

The table shows the mean values and between-group differences in the secondary outcomes at the 3, 6 and 12-month follow-ups with 95% confidence intervals. The trajectories are estimated using a mixed-effects model repeated measures (MMRM) ANOVA with adjustment for baseline imbalance. N denotes the number of subjects.

| Outcomes | Arthroscopic subacromial decompression (ASD) | n | Exercise Therapy (ET) | n | Between-group difference ASD vs. ET |
|---|---|---|---|---|---|
| **3 months** | | | | | |
| Constant-Murley score | N/A | | N/A | | N/A |
| SST | N/A | | N/A | | N/A |
| Satisfaction to the treatment (0-100 VAS) | 76.9 (71.6 to 82.3) | 57 | 79.2 (74.3 to 84.2) | 67 | -2.3 (-9.6 to 5.0) |
| 15-D | 0.9 (0.9 to 0.9) | 55 | 0.9 (0.9 to 0.9) | 64 | 0 (-0 to 0) |
| **6 months** | | | | | |
| Constant-Murley score | 59.2 (54.9 to 63.6) | 59 | 58.1 (54.1 to 62.2) | 67 | 1.1 (-4.8 to 7.1) |
| SST | 7.9 (7.3 to 8.5) | 59 | 7.9 (7.4 to 8.5) | 67 | 0 (-1.0 to 1.0) |
| Satisfaction to the treatment (0-100 VAS) | 74.9 (69.5 to 80.3) | 56 | 75.2 (70.2 to 80.2) | 66 | -0.3 (-7.6 to 7.1) |
| 15-D | 0.9 (0.9 to 0.9) | 55 | 0.9 (0.9 to 0.9) | 64 | 0 (-0 to 0) |
| **12 months** | | | | | |
| Constant-Murley score | N/A | | N/A | | N/A |
| SST | N/A | | N/A | | N/A |
| Satisfaction to the treatment (0-100 VAS) | 82.9 (77.4 to 88.3) | 55 | 78.1 (73.1 to 83.2) | 64 | 4.7 (-2.7 to 12.1) |
| 15-D | 0.9 (0.9 to 0.9) | 54 | 0.9 (0.9 to 0.9) | 62 | 0 (-0 to 0) |

The between-group differences may not exactly equal the differences in change in the scores between the ASD and DA groups because of the adjustment for baseline imbalance in the MMRM analyses. N/A = not applicable. For all variables, a higher score indicates a better treatment outcome.

**Table S10 Sensitivity analyses of the primary and secondary outcomes concerning the secondary comparison (ASD vs. ET).**
The table shows the between-group differences at the 24-month follow up. The estimated effect indicates the mean difference, ASD minus ET. The analyses were carried out using a mixed model repeated measures (MMRM) ANOVA with adjustment for baseline imbalance.

| Variable | FAS Estimated effect (95% CI)* | P value | Per protocol Estimated effect (95% CI)† | P value | As treated Estimated effect (95% CI)‡ | P value | Worst case Estimated effect (95% CI)§ | P value |
|---|---|---|---|---|---|---|---|---|
| Pain VAS at rest | -7.52 (-14.02 to -1.02) | 0.02 | -9.62 (-16.19 to -3.05) | 0.004 | -9.36 (-15.76 to -2.96) | 0.004 | -8.57 (-15.26 to -1.88) | 0.012 |
| Pain VAS on activity | -12.02 (-20.87 to -3.17) | 0.008 | -12.11 (-21.35 to -2.87) | 0.010 | -8.66 (-17.41 to 0.09) | 0.053 | -11.23 (-20.24 to -2.22) | 0.014 |
| Constant-Murley score | 7.73 (1.61 to 13.85) | 0.013 | 6.10 ( -0.18 to 12.38) | 0.057 | 2.90 (-3.14 to 8.94) | 0.35 | 4.74 (-2.14 to 11.62) | 0.177 |
| SST | 0.66 (-0.16 to 1.48) | 0.12 | 0.71 ( -0.15 to 1.57) | 0.11 | 0.41 (-0.41 to 1.23) | 0.33 | 0.58 (-0.41 to 1.56) | 0.251 |
| Satisfaction to treatment (0-100 VAS) | 3.31 (-3.87 to 10.49) | 0.37 | 3.15 ( -4.09 to 10.39) | 0.97 | 1.74 (-5.30 to 8.78) | 0.63 | 3.35 (-3.97 to 10.68) | 0.369 |
| 15-D | 0.00 (-0.02 to 0.02) | 1.00 | 0.01 ( -0.01 to 0.03) | 0.32 | 0.01 (-0.01 to 0.03) | 0.32 | 0.00 (-0.02 to 0.03) | 0.800 |

* FAS, the full analysis set population (ASD: n = 59, ET: n = 71).

† The per protocol population is the subset of the full analysis set population who received the treatment they were randomised to and who did not receive any other treatment, i.e. the patients with a treatment conversion have been excluded (ASD: n = 59, ET: n = 57).

‡ The as treated population is defined according to the treatment the participants received, i.e., the 8 participants who originally received DA and the 14 participants who originally received ET, but due to persistent symptoms requested unblinding and subsequently received ASD, have been included in the ASD population (ASD: n = 81, ET: n = 57).

§ The worst-case population is a subset of the full analysis set population in which seven participants in the ET group have been removed due to comparable pathology as in the participants who were excluded from the surgical groups (ASD and DA) before the second randomisation. The exclusion of the participants in the ET group was based on that they had received surgical treatment due to reasons other than impingement during the follow-up (ASD: n = 59, ET: n = 64).

**Table S11 Proportions of patients in the ASD and ET groups exceeding the threshold for minimal clinically important improvement (MCII) and reaching the patient-acceptable symptom state (PASS).**

| Variable | ET (n=68) | ASD (n=59) | Chi-square P value |
|---|---|---|---|
| **Proportion of patients exceeding the MCII*** | **n (%)** | **n (%)** | |
| Pain VAS at rest | 42 (62) | 45 (76) | 0.079 |
| Pain VAS on activity | 53 (78) | 49 (83) | 0.47 |
| **Proportion of patients reaching the PASS†** | | | |
| Pain VAS at rest | 60 (88) | 56 (95) | 0.18 |
| Pain VAS on activity | 43 (63) | 48 (81) | 0.024 |

* An improvement of at least 15 points from baseline value was required to consider an individual reaching the minimal clinically important improvement (MCII) in VAS.

† The cut-off limit for reaching the patient acceptable symptomatic state (PASS) in VAS (0 – 100 scale) at 24 months was set at <30 points.

**Table S12 Frequency of missing data at 3, 6, 12 and 24 months.**

| Study group | Timepoint | Pain VAS at rest | Pain VAS on activity | Constant-Murley score | SST | 15D |
|---|---|---|---|---|---|---|
| ASD (n=59) | Baseline | 0 | 0 | 0 | 0 | 2 |
| | 3 months | 5 | 7 | N/A | N/A | 2 |
| | 6 months | 0 | 0 | 0 | 0 | 3 |
| | 12 months | 4 | 4 | N/A | N/A | 3 |
| | 24 months | 0 | 0 | 1 | 1 | 1 |
| DA (n=63) | Baseline | 0 | 0 | 0 | 0 | 3 |
| | 3 months | 8 | 8 | N/A | N/A | 6 |
| | 6 months | 2 | 2 | 2 | 2 | 5 |
| | 12 months | 7 | 7 | N/A | N/A | 8 |
| | 24 months | 4 | 4 | 4 | 4 | 5 |
| ET (n=71) | Baseline | 1 | 1 | 1 | 1 | 2 |
| | 3 months | 9 | 9 | N/A | N/A | 6 |
| | 6 months | 3 | 3 | 3 | 3 | 5 |
| | 12 months | 6 | 7 | N/A | N/A | 7 |
| | 24 months | 3 | 3 | 6 | 5 | 3 |

Abbreviations: ASD = Arthroscopic Subacromial Decompression; DA = Diagnostic arthroscopy; ET = Exercise therapy; VAS = Visual analogue scale SST = Simple Shoulder Test.

The n values denote the number of participants allocated to each treatment group. The frequencies include both missing data and withdrawn subjects.
